# GABA/Glutamate Neuron Differentiation Imbalance and Increased AKT/mTOR Signaling in CNTNAP2^−/−^ Cerebral Organoids

**DOI:** 10.1016/j.bpsgos.2024.100413

**Published:** 2024-11-08

**Authors:** Kleanthi Chalkiadaki, Elpida Statoulla, Maria Zafeiri, Georgia Voudouri, Theoklitos Amvrosiadis, Alexandra Typou, Niki Theodoridou, Dimitrios Moschovas, Apostolos Avgeropoulos, Martina Samiotaki, John O. Mason, Christos G. Gkogkas

**Affiliations:** a Biomedical Research Institute, Foundation for Research and Technology-Hellas, University Campus, Ioannina, Greece; b Centre for Discovery Brain Sciences, University of Edinburgh, Edinburgh, United Kingdom; c Department of Materials Science Engineering, University of Ioannina, Ioannina, Greece; d Biomedical Sciences Research Center “Alexander Fleming”, Vari, Greece; e Simons Initiative for the Developing Brain, University of Edinburgh, Edinburgh, United Kingdom

**Keywords:** AKT/mTOR, Autism, Cerebral organoids, CNTNAP2, GABA/glutamate, Neurodevelopment

## Abstract

**Background:**

The polygenic nature of autism spectrum disorder (ASD) requires the identification of converging genetic pathways during early development to elucidate its complexity and varied manifestations.

**Methods:**

We developed a human cerebral organoid model from induced pluripotent stem cells with targeted genome editing to abolish protein expression of the *CNTNAP2* ASD risk gene.

**Results:**

CNTNAP2^−/−^ cerebral organoids displayed accelerated cell cycle, ventricular zone disorganization, and increased cortical folding. Proteomic analysis revealed disruptions in glutamatergic/GABAergic (gamma-aminobutyric acidergic) synaptic pathways and neurodevelopment, and transcriptomic analysis revealed differentially expressed genes belonging to inhibitory neuron–related gene networks. Interestingly, there was a weak correlation between the 2 datasets, suggesting nuanced translational control mechanisms. Along these lines, we found upregulated AKT/mTOR (mechanistic target of rapamycin) signaling in CNTNAP2^−/−^ organoids. Spatial transcriptomic analysis of CNTNAP2^−/−^ ventricular-like zones demonstrated pervasive changes in gene expression, implicating upregulation of cell cycle regulation, synaptic, and glutamatergic/GABAergic pathways. We noted significant overlap of all day-30 organoid omics datasets differentially expressed genes from idiopathic ASD (macrocephaly) induced pluripotent stem cell–derived telencephalic organoids, where FOXG1 was upregulated. Moreover, we detected increased GAD1-expressing and decreased TBR1-expressing cells, suggesting altered GABAergic/glutamatergic neuron development.

**Conclusions:**

These findings potentially highlight a shared mechanism in the early cortical development of various forms of ASD, further elucidate the role of CNTNAP2 in ASD pathophysiology and cortical development, and pave the way for targeted therapies that use cerebral organoids as preclinical models.

## Plain Language Summary

Chalkiadaki *et al.* developed a human cerebral organoid model by using genome-edited induced pluripotent stem cells to study autism spectrum disorder (ASD). The model, which lacks the *CNTNAP2* gene associated with ASD, showed abnormal brain development, including disrupted neuron differentiation and increased signaling in pathways linked to neurodevelopment. These findings suggest that CNTNAP2 plays a critical role in early brain formation and may contribute to ASD pathophysiology, thus providing a platform for exploring potential therapies. The study highlights the importance of CNTNAP2 in neuron development and its impact on cortical structure in ASD.

Elucidating the polygenic basis of neurodevelopmental disorders remains an important challenge (1). Autism spectrum disorder (ASD) is a complex neurodevelopmental condition characterized by challenges in social interaction, communication, and a tendency toward repetitive behaviors and restricted interests (2). The advent of human induced pluripotent stem cell (iPSC)–derived brain organoids offers a versatile platform to elucidate the molecular and cellular mechanisms that are implicated in brain development and study genetic and pharmacological models where development can go awry as in ASD, ultimately linking basic research to the development of therapies or to personalized medicine applications (3).

The *CNTNAP2* gene is one of the largest genes in the human genome, spanning approximately 2.3 million base pairs (4). The CNTNAP2 (or CASPR2) protein belongs to the neurexin family and is a transmembrane protein integral to the function of juxtaparanodes in myelinated neurons (5). It facilitates the organization of key proteins like TAG-1 and Kv.1, which regulate neuronal excitability (6). Highly penetrant, homozygous loss-of-function mutations in *CNTNAP2* lead to cortical dysplasia focal epilepsy syndrome, which is marked by ASD, intellectual disability, and epilepsy, underscoring its critical role in brain development and function (7). Importantly, CNTNAP2 has been linked to cortical interneuron development (8), which in turn has been implicated in the pathophysiology of ASD (9). Loss of CNTNAP2 results in abnormal neuronal migration and altered distribution of cortical GABAergic (gamma-aminobutyric acidergic) interneurons in mouse and zebrafish (8,10). On the other hand, loss of CNTNAP2 has been shown to reduce neurite branching and overall neuronal complexity in developing human excitatory neurons (11). Core phenotypes in *Cntnap2*^*−/−*^ mice were reversed with oxytocin administration (12). Moreover, *Cntnap2* deletion led to activation of Akt/mTOR (mechanistic target of rapamycin) signaling in the mouse brain (13). Pharmacological inhibition of this pathway reversed core autism-related phenotypes in *Cntnap2*^*−/−*^ mice (13). In addition, recent studies have revealed that human cortical development is heavily reliant on posttranscriptional regulatory mechanisms, particularly through the regulation by mTOR. mTOR signaling has been shown to regulate the architecture of the developing human cortex by maintaining the cytoskeletal organization in outer radial glial cells and the stability of the radial glia scaffold (14). Furthermore, regulation of translation in early human cerebral organoid progenitor cells, particularly of the 5′ terminal oligopyrimidine tract element–enriched messenger RNAs (mRNAs), is mediated by mTOR (15). A recent study that used forebrain organoids derived from patient iPSCs harboring *CNTNAP2* c.3709DelG revealed cortical overgrowth and aberrant cellular proliferation because of *CNTNAP2* loss of function (16). However, our understanding of the mechanisms downstream of *CNTNAP2* that go awry in neurodevelopmental disorders such as ASD is incomplete.

Herein, we used a human cerebral organoid model derived from a commercially available iPSC line where CNTNAP2 protein expression was abolished via targeted genome editing to mimic loss-of-function mutations in patients with ASD and study mechanisms that underlie early cortical development, using proteomics, transcriptomics, and spatial transcriptomics.

## Methods and Materials

The full methods description is provided in the Supplement.

### iPSCs Generation

We used the commercially available iPSC lines XCL1 and XCL1-*CNTNAP2*^*−/−*^ (XCell Science). The biallelic CNTNAP2 knockout (KO) line was generated with the Zinc finger nuclease method (Figure S1A). All experiments involving iPSCs were approved by the FORTH Ethics and Deontology Committee.

### Cell Culture and Cerebral Organoid Generation

iPSC lines were maintained at 37 °C with 5% CO_2_ in mTeSR Plus on Matrigel-coated plates. Cerebral organoids were generated using a modified organoid differentiation protocol (17,18) and were maintained on orbital shakers for 60 days. Half medium changes were performed every other day.

### Proteomics Sample Preparation

For each biological replicate, 3 to 4 day 30 (D30) to D33 organoids were pooled and lysed together. The samples were sonicated and heated for 3 minutes at 95 °C followed by 15-minute centrifugation at 17,000*g*. Lysed samples were processed with Sp3 protocol (19) including an alkylation step in 200 mM iodoacetamide.

### Liquid Chromatography Tandem Mass Spectrometry and Analysis

Samples were processed with liquid chromatography tandem mass spectrometry setup. Analysis was performed according to (20) and (21).

### EdU Click-It Assay

D30 organoids were incubated in 10 μM EdU diluted in cerebral organoid differentiation medium for 2 hours and subsequently collected and processed. EdU click-it assay was performed in organoid cryosections per manufacturer’s instructions followed by immunostaining for Ki67.

### RNA Sequencing and Bioinformatics Analysis

For each biological replicate, 3 to 4 D30 organoids were pooled and homogenized using QIAshredder homogenizers. Total RNA was extracted using the RNeasy Micro kit per manufacturer’s instructions. Library preparation and RNA sequencing (RNAseq) were performed as a service by GENEWIZ/AZENTA and sequenced on a Novaseq 6000 instrument (Illumina).

### Spatial Transcriptomics With GeoMx and Bioinformatics Analysis

Spatial transcriptomics was conducted using the NanoString GeoMx Digital Spatial Profiler in D30 cerebral organoids. PAX6, NESTIN, and SYTO83 antibodies were used to define areas of interest. Samples were processed with the GeoMx DSP instrument, followed by library preparation and sequencing on an Illumina NovaSeq 6000 system. Data were processed and analyzed on the GeoMx DSP online platform. Differential expression analysis compared PAX6^−^NESTIN^+^ and PAX6^+^NESTIN^+^ cells in both control and KO groups, as well as KO versus control in these cell types.

### Image Analysis

Bright-field images were captured on an EVOS XL Core microscope. Organoid surface area was quantified in ImageJ. Fold density was measured using the Canny edge detection plug-in in ImageJ (22). Cell counting was done manually for the EdU assay or using ImageJ [see (23)].

## Results

### CNTNAP2 Targeted Deletion Alters Early Cortical Development in Human iPSC-Derived Cerebral Organoids

**Figure 1:**
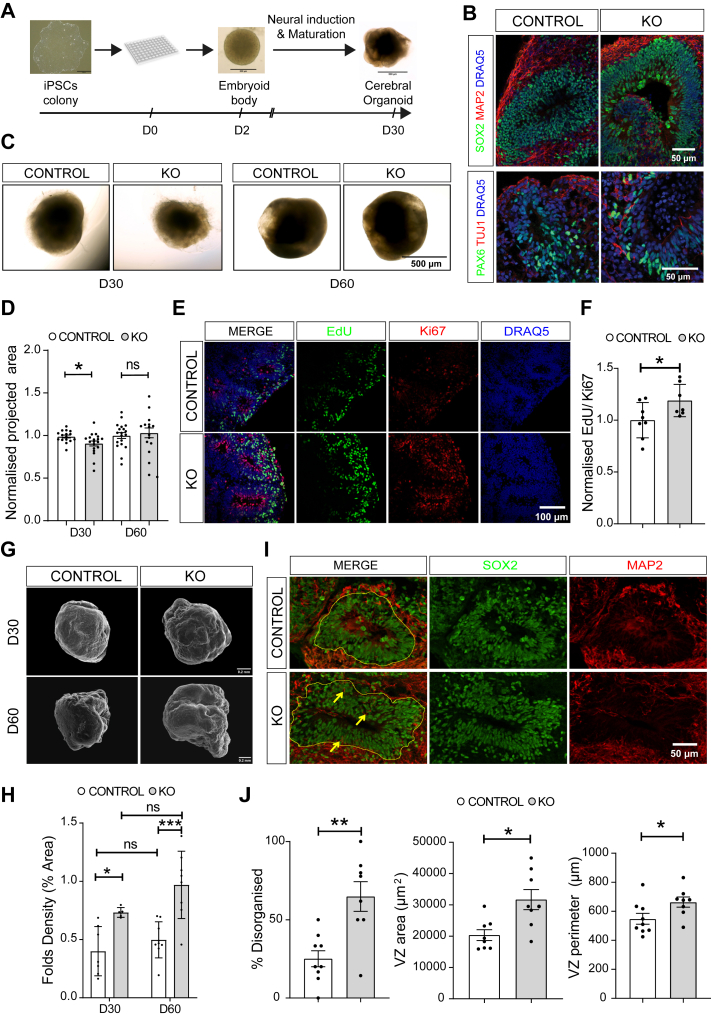
CNTNAP2 targeted deletion alters early cortical development in iPSC-derived cerebral organoids. **(A)** Schematic overview of the cerebral organoid protocol development until day 30 of differentiation. **(B)** Representative images from D30 control and KO organoids showing expression of the NPC markers SOX2 and PAX6 and the neuronal markers TUJ1 and MAP2. **(C)** Representative bright field images from control and KO organoids. **(D)** Normalized projected surface area measurement showing a decrease in size in KO organoids at D30, which is eliminated at D60 of differentiation (*n* = 19 for control D30, control D60, and KO D30 and *n* = 16 for KO D60, 2 separate organoid batches per genotype for each age group). **(E)** Representative images from EdU and Ki67 immunostaining of D30 organoids. **(F)** Bar graph showing increased EdU/Ki67 immunofluorescence signal ratio in KO organoids (*n* = 7/group, 3 separate organoid batches per genotype, KO values are normalized to the control mean). Quantification of cell cycle length was performed using the formula Tc = Ts/(EdU^+^/Ki67^+^) as previously described (16). KO organoids showed shorter cell cycle length than control organoids. **(G)** Representative scanning electron microscopy images from control and KO organoids. **(H)** Quantification of surface fold density showing increased surface folding in KO-derived organoids at D30 and D60 of differentiation (*n* = 7 for control D30, *n* = 5 for KO D30, *n* = 9 for control D60, and *n* = 8 for KO D60, 2 separate organoid batches per genotype for each age group). **(I)** Representative images from control and KO organoids with SOX2 (green) and MAP2 (red) immunostaining, demonstrating increased size and disorganization of the VZ-like structures in KO organoids. The yellow line delineates the VZ region, and yellow arrows emphasize the presence of MAP2-positive neurons inside the VZ. **(J)** Quantification of the area and perimeter of VZ and the occurrence of organized vs. disorganized VZ regions in control and KO organoids, based on SOX2 and MAP2 staining patterns (*n* = 9 for control and *n* = 8 for KO organoids, 4 separate organoid batches per genotype). For panels **(D)**, **(F)**, and **(J)**, Student’s *t* test analysis, ∗*p* < .05, ∗∗*p* < .01. For panel **(H)**, 2-way ANOVA with Tukey’s post hoc test, organoids age: *F*_1,25_ = 4.596, *p* = .042, genotype: *F*_1,25_ = 26.59, *p* < .0001, interaction: *F*_1,25_ = 0.791, *p* = .382, ∗∗∗*p* < .001. Also see Figure S1 and Table S7. ANOVA, analysis of variance; D, day; iPSCs, induced pluripotent stem cells; KO, knockout; NPC, neural progenitor cell; ns, not significant; VZ, ventricular zone.

To study the role of CNTNAP2 in early cortical development, we obtained *CNTNAP2*^*−/−*^ iPSCs (KO) generated with Zinc finger targeted genome editing of both alleles in the XCL1 male human pluripotent stem cell line (control) (24) (Figure S1A). KO and isogenic XCL1 control iPSC displayed normal karyotype (CGH array) (Figure S1E) and were tested for pluripotency by measuring expression of OCT-3/4 and NANOG (with immunofluorescence and reverse transcriptase–quantitative polymerase chain reaction) (Figure S1F). Using control and KO cells, we derived cerebral organoids using a modified protocol (see Methods and Materials) from prior work (17, 18) (Figure 1A). Cerebral organoids expressed typical key markers of the early developing brain. Confocal imaging of immunofluorescence-labeled cerebral organoid slices at D30 showed prominent expression of the neural progenitor markers SOX2 and PAX6 proteins within the ventricular zone (VZ) structures in both genotypes. Conversely, more mature neurons expressing TUJ1 and MAP2 were located outside the VZ-like structures (Figure 1B). We detected a 90% reduction in *CNTNAP2* mRNA expression in KO iPSCs (nested *t* test, *p* < .0001) and no CNTNAP2 protein expression by immunoblotting in D30 KO organoids compared with control (Figure 1C, D). Then, we proceeded to analyze the morphology of organoids. First, using bright-field microscopy, we observed that D30 KO organoids were 8% smaller than control organoids (Mann-Whitney test, *p* = .024), but no significant size difference was seen at D60 (Student’s *t* test, *p* = .633) as evidenced by the projected surface area measurements (Figure 1C, D). Second, to assess the proliferation during early development of organoids, we performed cell-cycle length assessment by colabeling with EdU and the proliferating protein marker Ki67 coupled with confocal imaging (Figure 1E). D30 KO organoids displayed a higher EdU/Ki67 ratio (19%), suggesting increased proliferative potential of neural progenitor cells (NPCs) and shorter cell-cycle duration (Student’s *t* test, *p* = .043) (Figure 1E, F). Third, we proceeded to assess surface folding of cerebral organoids because expansion and cortical folding in the human brain relies on NPC proliferation. We used surface electron microscopy to image the gold/palladium-coated surface of D30 cerebral organoids and identified an 82% and 95% increase in surface folding density of D30 and D60 KO organoids, respectively (2-way analysis of variance with Tukey’s post hoc test, organoids age: *F*_1,25_ = 4.596, *p* = .042, genotype: *F*_1,25_ = 26.59, *p* < .0001, interaction: *F*_1,25_ = 0.791, *p* = .382) (Figure 1G, H). Fourth, to further validate the size and the surface folding alterations, we sectioned D30 organoids, and we assessed the size and organization of VZ-like structures, at the histological level. SOX2-positive NPCs and MAP2-positive neurons defined the VZ region boundaries. KO organoids showed significantly increased VZ area (56%, Student’s *t* test, *p* = .0076) and perimeter (21%, Student’s *t* test, *p* = .042) compared with control organoids, while the extensive presence of MAP2-positive cells within the VZ-like structure indicated a disruption in the organization of cells (Figure 1I, J). Disorganization of VZ-like structure cells could impact corticogenesis as has been shown for other ASD mutations (e.g., synaptic Ras GTPase activating protein 1 [SYNGAP1] or phosphatase and tensin homolog [PTEN]) modeled with brain organoids (22,25).

### Altered Proteomic Landscape in CNTNAP2^−/−^ Cerebral Organoids

**Figure 2:**
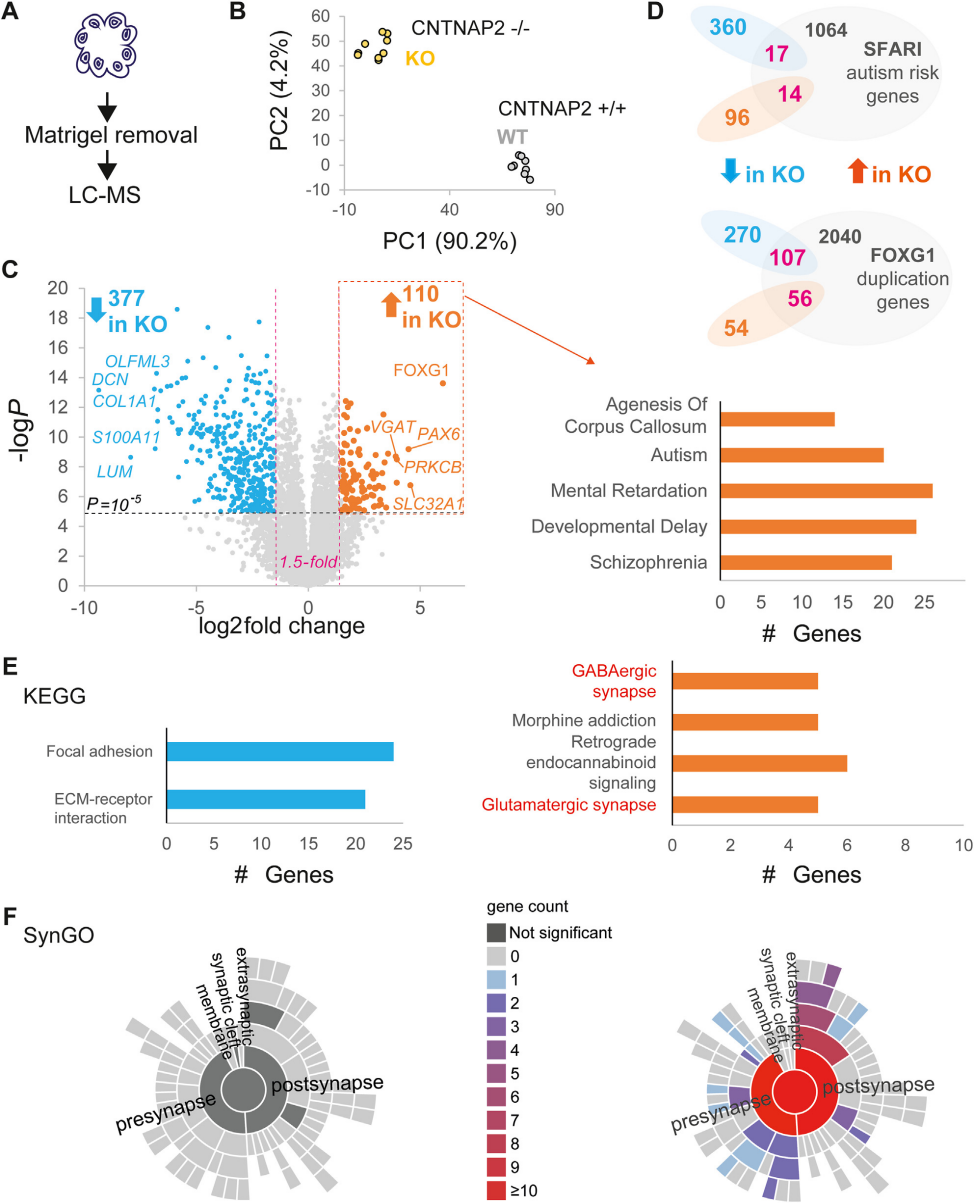
Altered proteomic landscape in CNTNAP2^−/−^ cerebral organoids. **(A)** Illustration of cerebral organoid Matrigel removal and lysis for LC-MS. **(B)** PCA for proteomics biological (*n* = 3) and technical (*n* = 3) replicates of CNTNAP2 control (+/+) or KO (−/−) organoids (1 organoid batch per genotype). Scatter plot visualizing the proportion of explained variance, with PC1 accounting for the majority (90.2%) of the variance. PC2 explains a smaller portion of the variance (4.2%). **(C)** Left: Volcano plot of D30 proteomics experiment highlighting upregulated (orange) and downregulated (cyan) peptides in KO samples. The x-axis demonstrates the log-transformed fold change in abundance (KO/control), and the y-axis indicates the log-transformed *p* values associated with individual peptides. A cutoff of ±1.5 fold-change (dashed vertical lines) and *p* value >10^−5^ (dashed horizontal line) was applied. Right: GO analysis of upregulated peptides in KO with the GeDiPNet database (Enrichr). **(D)** Venn diagrams showing overlap (magenta) of differentially expressed peptides in KO with top: SFARI syndromic ASD genes (https://gene.sfari.org/) and bottom: RNAseq data from (26) (FOXG1 upregulation in idiopathic ASD). **(E)** KEGG and **(F)** SynGO GO analysis of downregulated (left) and upregulated (right) peptides in KO cerebral organoids. Also see Figure S2 and Tables S1 and S2. ASD, autism spectrum disorder; D, day; ECM, extracellular matrix; GABA, gamma-aminobutyric acid; GO, Gene Ontology; KEGG, Kyoto Encyclopedia of Genes and Genomes; KO, knockout; LC-MS, liquid chromatography mass spectrometry; PCA, principal component analysis; RNAseq, RNA sequencing; SFARI, Simons Foundation Autism Research Initiative; SynGO, Synaptic Gene Ontologies; WT, wild-type.

To examine the mechanisms that underlie *CNTNAP2*^*−/−*^ cerebral organoid phenotypes, we performed label-free phosphoproteomic analysis of D30 CNTNAP2 control and KO cerebral organoids after Matrigel removal, using the Sp3-mediated protein digestion method (19) coupled with liquid chromatography–mass spectrometry. Using a data-independent acquisition mass spectrometry strategy (20) enabled the identification of 10,355 protein groups based on 111,568 tryptic peptides (Table S1). We proceeded with the statistical analysis of the proteome generated based on the proteotypic peptides (peptides unique for a protein), which numbered a total of 8232 proteins. To test for variation in biological and technical replicates, we performed principal component analysis and confirmed high reproducibility between KO and control samples (Figure 2B). Proteomic analysis in KO organoids revealed changes in a significant portion of peptides compared with control organoids (−1.5 > fold change > 1.5; false discovery rate < .01; *p* < 10^−5^; 377 downregulated and 110 upregulated unique peptides) (Figure 2C and Table S1). Because *CNTNAP2* is an autism risk gene, we compared this dataset to the SFARI (Simons Foundation Autism Research Initiative) autism gene database and confirmed 31 overlapping genes (Figure 2D). Among the top upregulated targets in KO proteomic analysis, we detected FOXG1, PAX6, PRKCB, and SLC32A1 (Figure 2C). Because FOXG1 was the top upregulated target in KO organoids, we compared the proteomics dataset to a recent RNAseq dataset from human telencephalic organoids derived from iPSCs from patients with idiopathic ASD, where *FOXG1* was upregulated (26), and detected significant overlap of 163 targets (Figure 2D). To further understand the pathways downstream of CNTNAP2 deletion in human cerebral organoids, we performed gene ontology (GO) analysis of genes coding for the differentially expressed peptides detected in the proteomics dataset (Table S2). GO analysis of the 110 upregulated genes showed significant enrichment of terms such as “nervous system development,” “synapse,” “neuron differentiation,” “neurogenesis,” and “generation of neurons,” while GO terms for the 377 downregulated genes were predominantly related to the extracellular matrix (ECM) (organization, adhesion, collagen) (Figure 2C, E and Table S2). Interestingly, upregulated genes in Kyoto Encyclopedia of Genes and Genomes and synaptic GO analysis highlighted terms such as GABAergic and glutamatergic synapses and pre- and postsynapses (Figure 2E, F). GO analysis in the GeDiPNet database (Enrichr) was enriched for terms such as: “agenesis of corpus callosum,” “autism,” “mental retardation,” “developmental delay,” and “schizophrenia” (Figure 2C). Conversely, the downregulated peptides GO dataset analysis showed ECM and focal adhesion pathways and no significant enrichment for synaptic or autism-related pathways (Figure 2E, F). We also analyzed D60 organoids with proteomics (Figure S2).

### Pro-Interneuron Transcriptional Networks in CNTNAP2^−/−^ Cerebral Organoids

**Figure 3:**
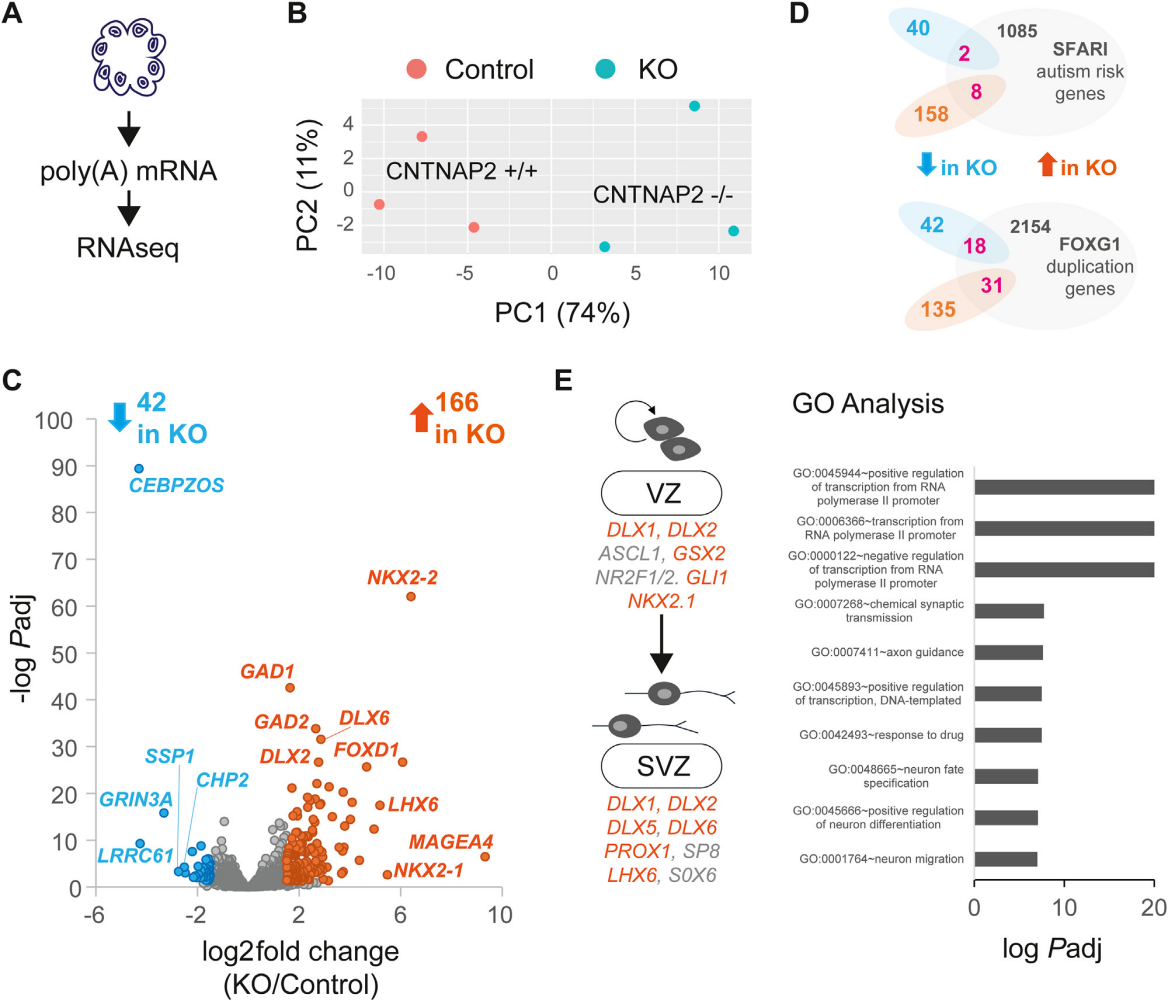
Pro-interneuron transcriptional networks in CNTNAP2^−/−^ cerebral organoids. **(A)** Illustration of cerebral organoid poly(A) mRNA isolation and RNAseq. **(B)** PCA for RNAseq biological (*n* = 3) replicates of CNTNAP2 control (^+/+^) or KO (^−/−^) organoids (1 organoid batch per genotype). Scatter plot visualizing the proportion of explained variance, with PC1 accounting for the majority (74%) of the variance. PC2 explains a smaller portion of the variance (11%). **(C)** Volcano plot of D30 RNAseq experiment highlighting upregulated (orange) and downregulated (cyan) DEGs in KO samples. The x-axis demonstrates the log_2_-transformed fold change in abundance (KO/control), and the y-axis indicates the negative log-transformed adjusted *p* values associated with individual mRNAs. A cutoff of ±1.5 fold-change (dashed vertical lines) and *p* value >10^−5^ (dashed horizontal line) were applied. **(D)** Venn diagrams showing overlap of DEGs in KO with top: SFARI syndromic ASD genes (https://gene.sfari.org/), and bottom: RNAseq data from (18) (FOXG1 upregulation in idiopathic ASD). **(E)** Left: Illustration of pro-interneuron mRNAs upregulated (in orange) in CNTNAP2 KO RNAseq DEGs, showing genes participating in VZ and SVZ neurogenesis, cell-fate commitment, and tangential migration (in gray). Right: GO analysis of DEGs using GeneSCF v1.1-p2. Significantly enriched GO categories are shown with adjusted *p* value <.05 (Fisher’s exact test). Also see Figure S3 and Tables S1 and S2. Adj, adjusted; D, day; DEG, differentially expressed gene; KO, knockout; mRNA, messenger RNA; PCA, principal component analysis; RNAseq, RNA sequencing; SVZ, subventricular zone; VZ, ventricular zone.

To further study changes in protein expression at the level of transcription in CNTNAP2^−/−^ cerebral organoids, we performed bulk RNA sequencing of mRNA extracted from D30 cerebral organoids (control or KO) (Figure 3A and Figure S3). Biological replicates were highly reproducible, as evidenced by the high correlation within genotypes in principal component analysis (Figure 3B). We detected 208 differentially expressed genes (DEGs), 42 downregulated and 166 upregulated (−log *p*_adjusted_ > 1.3, 1.5 < log_2_ fold change < −1.5) (Figure 3C), that overlapped with the SFARI (10 common genes) and FOXG1 upregulation (49 common genes) datasets (Figure 3D), but the degree of overlap is not as extensive as the overlap of the proteomics dataset with SFARI/FOXG1 datasets (Figure 2D). GO analysis showed enrichment for regulation of transcription, synaptic transmission, axon guidance, neuron fate specification, and neuron differentiation (Figure 3E). Notably, within the top DEGs, we detected upregulation of transcription factors associated with cortical interneuron development (such as distal-less homeobox [HOX] family genes, *DLX1* and *DLX2*, and *NKX2.1* and *LHX6*), which has been implicated in VZ neurogenesis and cell-fate commitment (27,28) and subventricular zone cell-fate commitment and tangential migration (29) (Figure 3E).

### Pervasive Changes in Gene Expression in PAX6^−^ Cells in CNTNAP2^−/−^ Brain Organoids

**Figure 4:**
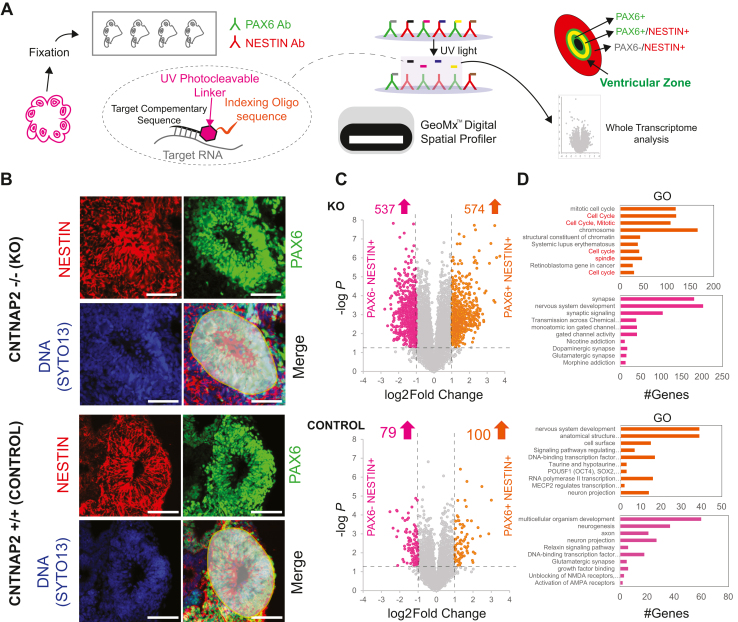
Spatial WTA shows changes in gene expression in PAX6^−^ cells in CNTNAP2^−/−^ brain organoids. **(A)** Illustration of experimental procedure to capture PAX6^+^ and NESTIN^+^ WTA with GeoMx (see text). **(B)** Representative images of immunostained slices from D30 control and KO cerebral organoids with GeoMx wide-field epifluorescence scope. Slices were stained for PAX6, NESTIN, and SYTO13 (a nuclear marker labeling DNA). Representative areas of interest captured (UV illumination) are shown in gray. **(C)** Left: Volcano plot of D30 spatial transcriptomics experiment highlighting upregulated and downregulated DEGs in KO (*n* = 3 biological replicates) or control samples (*n* = 3 biological replicates, 1 batch per genotype) (pink: PAX6^−^/NESTIN^+^, orange: PAX6^+^/NESTIN^+^). The x-axis demonstrates the log_2_-transformed fold change in abundance (KO/control), and the y-axis indicates the negative log-transformed adjusted *p* values associated with individual mRNAs. A cutoff of ±1 log_2_ fold-change (dashed vertical lines) and log *p* value >1.3 (dashed horizontal line) were applied. **(D)** GO of DEGs shown in **(C)** with g:Profiler. Top categories (biological process, molecular function, and cellular compartment) are shown for upregulated and downregulated DEGs in KO organoids. Statistical analysis was carried out using g:GOSt (Fisher’s 1-tailed test). Also see Figure 4 and Tables S5 and S6. Adj, adjusted; D, day; DEG, differentially expressed gene; GO, Gene Ontology; KO, knockout; mRNA, messenger RNA; WTA, whole-transcriptome analysis.

We employed spatial transcriptomics analysis using the NanoString platform GeoMx (Figure 4A) to measure whole-transcriptome changes in PAX6^+^ (neural progenitors) and PAX6^−^ cells (30). To ensure that we captured both cytoplasmic and nuclear RNAs, we used double labeling for PAX6 and NESTIN, respectively (Figure 4A, B). Sequencing saturation was >50%, and 13,457 genes normalized by third quartile, expressed above the limit of quantitation in at least 1% of areas of interest (Figure S4A–C). Initially, we compared PAX6^−^ and PAX6^+^ cells separately for each genotype (control or KO) (Figure 4C, D). Whole-transcriptome analysis of PAX6^−^/NESTIN^+^ cells versus PAX6^+^/NESTIN^+^ cells showed overall significantly altered mRNA expression (−log *p* > 1.2, 1 < log_2_ fold change < −1), which was more prominent in KO (1111 DEGs) than in control organoids (179 DEGs) (Figure 4C). PAX6^−^/NESTIN^+^ cell GO analysis was enriched for synaptic pathways in both genotypes (Figure 4D), and this effect was more pronounced in KO (537 DEGs) than in control (79 DEGs) organoids. Notably, we detected 574 DEGs in KO PAX6^+^/NESTIN^+^ (compared with PAX6^−^/NESTIN^+^ cells) with GO enrichment for cell cycle–related pathways (Figure 4D), consistent with the accelerated cell cycle of D30 cerebral organoids (Figure 1). In contrast, in control organoids PAX6^+^/NESTIN^+^ cells, the 100 DEGs were enriched for GO categories such as nervous system development and anatomical structure (Figure 4D).

Differential gene expression analysis in PAX6^−^/NESTIN^+^ cells highlighted significantly upregulated synaptic (glutamatergic/GABAergic) neurodevelopment-related pathways and pre-NOTCH transcription and translation and downregulated ECM-related pathways in KO compared with control organoids (Figure S4E). Notably, in PAX6^+^/NESTIN^+^ cells, differential gene expression analysis showed downregulation of ECM-related pathways and upregulation of HOX gene pathways in KO compared with control organoids (Figure S4F).

### Weak Correlation Between Proteomics and RNAseq and Altered AKT/mTOR Signaling in CNTNAP2^−/−^ Brain Organoids

**Figure 5:**
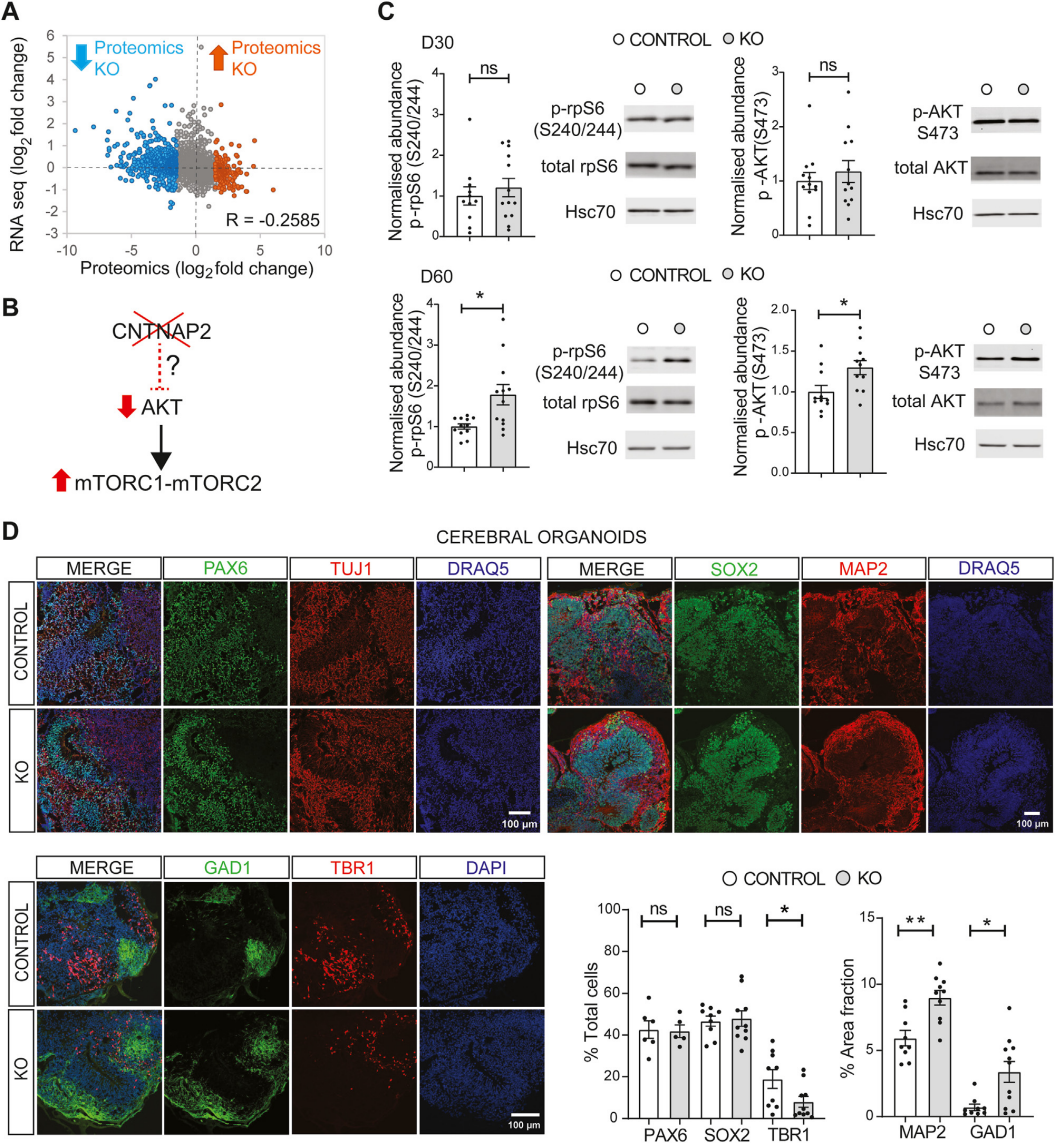
Weak correlation between proteomics and RNAseq, elevated AKT/mTOR signaling, and glutamatergic/GABAergic fate imbalance in CNTNAP2^−/−^ organoids. **(A)** Scatter plot showing the correlation coefficient of proteomics and RNAseq experiments (*R* = −0.2585); upregulated and downregulated peptides in KO from the mass spectrometry experiment (Figure 2) are shown in orange and cyan, respectively. **(B)** Illustration of possible mechanism for AKT/mTOR activation downstream of *CNTNAP2* loss of function. **(C)** Immunoblot analysis of AKT/mTOR signaling in D30 and D60 KO organoids. Left: Quantification of phospho-rpS6 (S240/244) and phospho-AKT (S473) for the indicated groups (*n* = 11–13/group, 4 separate organoid batches for D30, 3 separate organoid batches for D60, KO values are normalized to the control mean). Right: Representative immunoblots of cerebral organoids, probed with antisera against the indicated proteins. HSC70: loading control. **(D)** Representative images of immunostaining of SOX2^+^ and PAX6^+^ NPCs, TBR1^+^ postmitotic glutamatergic neurons and GAD1^+^ mature GABAergic interneurons, depicting the increased MAP2 and GAD1 expression in D30 KO cerebral organoids. Bottom right: Quantification of SOX2^+^, PAX6^+^, and TBR1^+^ cell fraction and MAP2 and GAD1 expression (% area fraction) in D30 cerebral organoids. Total cells were estimated by counting DAPI^+^ or DRAQ5^+^ nuclei (3–6 separate organoid batches per genotype, *n* = 1–3 organoids/batch). For **(C)** and **(D)**, Student’s *t* test, ∗∗*p* < .01, ∗*p* < .05. Also see Figure S5 and Table S7. D, day; GABA, gamma-aminobutyric acid; KO, knockout; NPCs, neural progenitor cells; ns, not significant; RNAseq, RNA sequencing.

Recent studies proposed that posttranscriptional regulatory mechanisms are required for the fidelity of cortical development and that this is largely due to mTOR regulation (14,15). Along the same lines, PTEN^−/−^ (phosphatase and tensin homolog, upstream of AKT/mTOR) cerebral organoids displayed increased AKT signaling and aberrant cortical development (22). Moreover, *Cntnap2*^*−/−*^ adult mice displayed increased Akt/mTOR signaling (13). First, we integrated D30 proteomics and transcriptomics datasets from control and KO cerebral organoids (Figure 5A). The correlation coefficients of log_2_ fold change in both experiments showed weak inverse correlation (*R* = −0.2585) (Figure 5A). Second, we measured AKT/mTOR signaling in cerebral organoids using immunoblotting for key effectors of this pathway: phospho-AKT S473 and phosphorylated rpS6 S240/244 (Figure 5C). While no significant changes were observed on D30, both phosphorylated AKT and phosphorylated rpS6 were increased (78% and 30%, respectively) in D60 KO cerebral organoids compared with control organoids (Student’s *t* test, *p* = .01; Mann-Whitney test, *p* = .023) (Figure 5C).

These data suggest that changes in mRNA levels do not consistently correlate with alterations in protein levels in CNTNAP2^−/−^ cerebral organoids, concomitant with upregulation of AKT/mTOR signaling.

### Glutamatergic/GABAergic Neuron Differentiation Imbalance in CNTNAP2^−/−^ Brain Organoids

Because FOXG1 upregulation was previously linked to glutamatergic/GABAergic neuron differentiation imbalance in iPSC-derived telencephalic organoids from patients with idiopathic ASD (26) and given the enrichment in GO terms linked to glutamatergic/GABAergic pathways in omics experiments (Figures 2 and 3), we reasoned that a similar mechanism may be at play in the CNTNAP2 model. GO analysis in all omics experiments revealed prominent glutamatergic and GABAergic pathways being regulated (Figure 2, Figure 3, Figure 4). To confirm this, we performed immunofluorescence analysis and confocal imaging in D30 control and KO cerebral organoids (Figure 5D) for markers of progenitors (SOX2, PAX6) and of more mature cells (MAP2, TBR1, GAD1). In KO compared with control organoids, we detected increased MAP2 and GAD1 expression (51% and 380%, respectively, Student’s *t* test, *p* = .0015; Mann-Whitney test, *p* = .016) (Figure 5D). Given the increased GAD1 expression, we further expanded the analysis and revealed prominent expression of the interneuron progenitor markers DLX2, NKX2.1, and GSH2 in D30 KO compared with control organoids (Student’s *t* test, *p* = .0355; Mann-Whitney test, *p* = .047; Mann-Whitney test, *p* = .035) (Figure S5). Conversely, we measured a decreased number of TRB1-positive cells (57%, Mann-Whitney test, *p* = .0435), but no changes in the number of SOX2- or PAX6-positive cells (Figure 5D), suggesting a shift toward GABAergic neuron progenitors in KO organoids.

## Discussion

Herein, the cerebral organoid model from iPSCs with targeted genome editing to knockout CNTNAP2 represents a significant advance in modeling the effects of *CNTNAP2* loss of function (Figure 1) using a commercially available iPSC line with homozygous targeted deletion of CNTNAP2 and an isogenic control line. Previous research utilized patient-derived iPSCs harboring CNTNAP2 mutations (16), and the work presented here offers an additional CNTNAP2 model that can be readily obtained, recapitulating key phenotypes that have been observed in patient-derived organoids. In our model, we did not observe significant changes in KO organoid size but report an increase in organoid surface folding (Figure 1), which is reminiscent of PTEN KO phenotypes (22). Malformations of cortical folding are common in ASD, epilepsy, and cortical focal dysplasias (31). As in (16), we also observed accelerated cell cycle and increased FOXG1 and PAX6 expression, highlighting the relevance and significance of the new model that we developed for studying *CNTNAP2* loss of function and ASD. Bulk RNAseq in D30 organoids revealed upregulation of pro-GABAergic interneuron fate transcriptional programs (Figure 3). Using spatial transcriptomics (Figure 4), we also revealed that CNTNAP2 deletion differentially affected PAX6^−^ and PAX6^+^ cells. Analysis showed upregulated cell cycle–related DEGs in KO PAX6^+^ cells, but not in control, compared with PAX6^−^ cells, where synaptic (glutamatergic/GABAergic) pathways were upregulated in both KO and control organoids. Little is known about the top downregulated DEG in KO: *CEBPZOS* coding for a mitochondrial protein related to energy metabolism (32). *CEBPZOS* downregulation in KO was further confirmed with RNAseq (Figure 4) and could be linked to GABAergic interneuron cell growth and apoptosis via its mitochondrial localization (33). Taken together, these cell type–specific and transcriptional effects further elucidate the role of CNTNAP2 in early cortical development and are consistent with the early maturation and glutamatergic/GABAergic neuron imbalance in CNTNAP2 KO organoids (Figure 5 and Figure S5).

We measured significant changes in protein expression (Figure 2) linked to neurodevelopmental, neurogenic, and synaptic pathways and significant changes in mRNA expression in D30 organoids (Figure 3) boosting pro-interneuron transcription programs in KO cerebral organoids. The weak correlation that we observed between transcriptomics and proteomics underscores the complexity of gene expression regulation in cortical progenitors (Figure 5) and was previously proposed as a new mechanistic explanation for aberrant corticogenesis in ASD (15). This finding highlights the crucial role of posttranscriptional and posttranslational mechanisms, particularly through the mTOR pathway, in regulating proteostasis, which necessitates deeper investigation.

Omics data generated with different methodologies (Figure 2, Figure 3, Figure 4) in this study showed significant overlap with RNAseq data from telencephalic organoids generated from iPSCs from patients with idiopathic ASD, where *FOXG1* was upregulated (26). FOXG1, a critical transcription factor, plays a key role in the development of cortical interneurons (34). FOXG1 suppresses the competence to generate the earliest-born neurons during later cortical development (35). *FOXG1* conditional deletion has been shown to impair the postnatal distribution of cortical interneurons, leading to enhanced dendritic complexity and decreased migration capacity (36). Interestingly, deletion of *Cntnap2* in mice influences the development and functional integration of interneurons (10), while CNTNAP2-regulated TAG1 is developmentally involved in axonal pathfinding (37). Analysis of P14 *Cntnap2* KO mice showed a decreased number of GABAergic interneurons (GAD1-, parvalbumin-, calbindin 2-, and neuropeptide Y-positive cells) in the somatosensory cortex and striatum (10). Similar GABAergic deficits (decreased DLX5a/6a-positive cells) were reported in the forebrain of Cntnap2 KO zebrafish (8). However, a recent study with early postnatal *Cntnap2* KO mice revealed an increased number of LHX6-positive striatal interneurons at postnatal day 0 (P0) and P4, which was restored at P6 (38). Consistent with this study, we found increased GAD1 expression (Figures 3C and 5D) in D30 KO organoids. Histological analysis further confirmed the altered GABAergic pool of neuronal progenitors in D30 KO organoids (increased DLX2-, NKX2.1-, and GSH2-positive cells) (Figure S5). GABAergic progenitor overproduction was also observed in 1-month-old human telencephalic organoids derived from iPSCs from patients with idiopathic ASD, where FOXG1 was upregulated (26), while ectopic interneuron generation was shown in 2-month cerebral organoids lacking PAX6 expression (17). However, in a previous report where human forebrain organoids (8-week-old) were generated from patient-derived iPSCs harboring *CNTNAP2* mutations, no changes were detected in interneuron development (16). This discrepancy could be attributed to the different developmental stages analyzed or the different protocols used for organoid generation. Nevertheless, ASD studies that focus on early neurogenesis, utilizing brain organoids, could be invaluable in unraveling the mechanisms and disrupted pathways that cause the disease and thus allow for early diagnosis and the design of new therapies.

Activation of pro-interneuron gene expression following *CNTNAP2* deletion, coupled with AKT/mTOR pathway hyperactivation, indicates a potentially key alteration in early neurodevelopmental processes (Figure 5). This finding suggests that the loss of CNTNAP2 leads to increased expression of transcription factors (such as DLX1/2, NKX2.1, and LHX6), which are known to shape VZ neurogenesis, cell-fate commitment, and subventricular zone tangential migration (27, 28, 29). Zebrafish and mouse *Cntnap2* KO models displayed changes in GABAergic interneuron migration (8,10). Hyperactivation of the AKT/mTOR pathway downstream of *CNTNAP2* loss of function, shown herein for a human model, is consistent with *Cntnap2*^*−/−*^ mouse model results (13) and could be critical for GABAergic interneuron fate determination and synaptic regulation. An absence of AKT/mTOR hyperactivation in D30 KO organoids (Figure 5) could be the result of a diluted or masked phenotype due to the bulk Western blot analysis. Plausibly, mTOR upregulation happens in a subset of more mature cells, which are more abundant on D60 and thus can be detected with Western blotting. Activation of the FOXG1/AKT/REELIN axis was previously shown in focal malformations of cortical development, whereby FOXG1-dependent derepression of *REELIN* transcription led to misexpression and non–cell autonomous defects in cell migration (39). Plausibly, this aberrant activation may also occur in other forms of syndromic or sporadic ASD in which there is AKT/mTOR [PTEN (22), TSC (15), SYNGAP1 (25)] or FOXG1 [idiopathic ASD (26)] upregulation.

Furthermore, spatial transcriptomics revealed additional transcriptional interplay involving HOX and NOTCH pathways upregulated in CNTNAP2 KO (Figure 4). HOX genes (*HOXD1*) map into human chromosome 2 region (2q31–2q33), where *GAD1* and *DLX2* also reside, and while linkage to autism is weak for the genes in this region (e.g., *GAD1*) (40), the sheer proximity could potentially signify transcriptional coregulation. In parallel, PAX6 homozygous deletion in human iPSCs led to upregulation of NOTCH and GABAergic interneuron-related transcriptional programs (DLX1/2/5/6, GSX2, GAD1/2) (17). NOTCH affects the proliferation/differentiation balance, and DLX and GSX2 govern interneuronal cell-fate acquisition. Thus, *CNTNAP2* loss of function may engender transcriptional dysregulation, which is critical for cortical proliferation and differentiation and the maintenance of the glutamatergic/GABAergic progenitor pool balance. FOXP2, the first gene linked to language and speech development (41), directly binds to regulatory regions of the *CNTNAP2* locus to repress its expression (42) and was shown to regulate both excitatory and inhibitory neuron development (43). Interestingly, TBR1 was shown to interact with FOXP2, and TBR1 mutations linked to sporadic ASD disrupt this interaction (44). Therefore, pro-interneuronal gene expression seen with RNAseq (Figure 4), in conjunction with the reduced number of TBR1 cells in CNTNAP2 KO organoids (Figure 5), could be the result of FOXP2/CNTNAP2/TBR1-mediated regulation. However, the discordance between transcriptomics and proteomics datasets (Figure 5) reveals another facet of regulation of gene expression and protein abundance downstream of *CNTNAP2* loss of function. This could relate to AKT/mTOR hyperactivation and translational control and operate in parallel to or independently of the transcriptional control via CNTNAP2.

In both mouse models (45) and human studies (46), CNTNAP2 has been linked to the size, structure, or connectivity of the corpus callosum, but the exact mechanisms by which CNTNAP2 affects the corpus callosum are still unclear. FOXG1 inactivation causes cerebral cortical hypoplasia and corpus callosum hypogenesis (47,48). Thus, it is conceivable that FOXG1 and CNTNAP2 share developmental roles regarding the proper development of the corpus callosum that involve regulation of gene expression via AKT/mTOR or FOXG1-mediated transcriptional programs.

### Study Limitations

We used 1 KO iPSC line (from XCL1 iPSC; male), which replicated most forebrain organoid phenotypes seen in patient iPSC lines (16). However, due to variability in iPSCs culture and differentiation (49), it is essential to study more clones or create additional CNTNAP2 loss-of-function mutants (e.g., via CRISPR/Cas9) in female iPSCs or other lines beyond XCL1 (11,16).

## Supplementary Material

Document S1 Supplemental Methods, Discussion, Figures S1–S5, and Table S7

Key Resources Table
