# Supplementary material for: GABA/Glutamate Neuron Differentiation Imbalance and Increased AKT/mTOR Signaling in CNTNAP2−/− Cerebral Organoids
Source: Biol Psychiatry Glob Open Sci. 2024 Nov 8;5(1):100413. doi: 10.1016/j.bpsgos.2024.100413 (PMC11699409; doi:10.1016/j.bpsgos.2024.100413)
Supplement: Document S1 — Supplemental Methods, Discussion, Figures S1–S5, and Table S7 [file mmc1.pdf]

SUPPLEMENTARY INFORMATION

GABA/Glutamate Neuron Differentiation Imbalance and Increased AKT/mTOR Signaling in CNTNAP2$^{-/-}$ Cerebral Organoids

Chalkiadaki *et al.*

<u>Contents</u>

Extended Methods and Materials

Study Limitations

Supplementary Figures 1-5

Supplementary Table 7 Statistical Analysis Details.

<u>See separate files for:</u>

Supplementary Table 1 Differentially expressed peptides in D30 and D60 cerebral organoids proteomics.

Supplementary Table 2 Gene Ontology analysis in D30 and D60 cerebral organoids proteomics.

Supplementary Table 3 Differentially expressed genes in D30 cerebral organoids RNAseq.

Supplementary Table 4 Gene Ontology analysis in D30 cerebral organoids RNAseq.

Supplementary Table 5 Differentially expressed genes in D30 cerebral organoids spatial transcriptomics.

Supplementary Table 6 Gene Ontology analysis in D30 cerebral organoids spatial transcriptomics.

Supplementary Figure 6 Uncropped immunoblot images.

Extended Methods and Materials

iPSC generation

We used the commercially available iPSC lines XCL1 and XCL1-*CNTNAP2*$^{-/-}$ (XCell Science). The bi-allelic CNTNAP2 KO line was generated with the Zinc finger Nuclease (ZFN) method (Sup. Fig. 1A). An insertion of 4bp and a deletion of 2bp were introduced in allele 1 and 2 of *CNTNAP2* gene in exon 7, respectively. Validation screening for CNTANP2 mutations was performed by Sanger sequencing and pluripotency tests were frequently performed, by measuring the expression of pluripotency markers (OCT-3/4 and NANOG) with qPCR and fluorescent Immunocytochemistry (ICC) (Sup. Fig. 1F). Karyotype analysis was conducted every ~10 passages using the Comparative Genome Hybridisation (CGH) array method (Sup. Fig. 1E). All experiments performed involving iPSCs were approved by FORTH Ethics and Deontology Committee

Cell culture and cerebral organoids generation

iPSC lines were maintained at 37^{0}C with 5% CO_2 in mTSER Plus (STEMCELL Technologies, #05825) on Matrigel® (Corning, #354277) coated plates and passaged using 0.5mM EDTA (Thermo Fisher Scientific, #15575020). Cerebral organoids were generated using a modified organoid differentiation protocol(1, 2). Briefly, 80% confluent iPSC colonies were dissociated into single cells with Accutase (Sigma-Aldrich, #A6964). A total of 9,000 cells were plated in each well of ultra-low attachment 96-well U-bottom plate (Corning, #7007). Embryoid bodies (EBs) were formed within the next 24 hours (day 1) and kept in the 96 well-plate for 6 days. EB medium changes were performed on day 2 (D2) and day 4. On D6, EBs were transferred to ultra-low attachment 24-well plates (Corning, #3473) and the medium was switched to neural induction medium with the addition of 10μM SB431542 (Selleck Chemicals, #S1067) and 100nM LDN-193189 (Selleck Chemicals, #S2618). On D10 EBs were embedded on Matrigel GFR® (Corning, #354230) droplets and cultured in cerebral organoid differentiation medium. Embedded EBs were kept on stationary culture for 24hrs, followed by transfer to an orbital shaker (Heathrow Scientific, #5003396). In order to aid neuronal maturation, 20ng/ml BDNF (Peprotech, #450-02) and NT3 (Peprotech, #450-03) was added to the medium at D30, for two weeks(1). Organoids were maintained on shakers for 60 days and half medium changes were performed every other day.

Proteomics sample preparation using the Sp3-mediated protein digestion protocol.

D30-D33 organoids were transferred from culture plates, briefly washed in ice-cold DPBS (PAN-Biotech, #P04-36500), depleted of Matrigel using the cell recovery solution (Corning #354253), supplemented with protease and phosphatase inhibitors, and subsequently lysed in a lysis buffer consisting of 4% SDS, 0.1M DTT, 0.1M Tris pH 7.4. For each biological replicate, 3-4 organoids were pooled and lysed together. The samples were sonicated (Digital Sonifier 250 Marshall Scientific), heated for 3 min at 95^{0}C, followed by a centrifugation step for 15 min at 17000 x g. The lysed samples were processed according to the Sp3 protocol(3) including an alkylation step in 200 mM iodoacetamide (Acros Organics). 20 ug of beads (1:1 mixture of hydrophilic and hydrophobic SeraMag carboxylate-modified beads, GE Life Sciences) were added to each sample in 50% ethanol. Protein clean-up was performed on a magnetic rack. The beads were washed two times with 80% ethanol and once with 100% acetonitrile (Fisher Chemical). Proteins captured on beads were digested overnight at 37^{0}C under vigorous shaking (1200 rpm, Eppendorf Thermomixer, #5382) supplemented with 0.5 μg Trypsin/LysC (MS grade, Promega) prepared in 25 mM ammonium bicarbonate. Next day, the supernatants were collected, and the peptides were purified using a modified Sp3 clean up protocol and finally solubilized in the mobile phase A (0.1% formic acid in water), sonicated and the peptide concentration was determined through absorbance at 280 nm measurement using a Nanodrop instrument (Thermo Fisher Scientific, Nanodrop One C).

LC-MS/MS.

Samples were processed on a liquid chromatography tandem mass spectrometry (LC-MS/MS) setup consisting of a Dionex Ultimate 3000RSLC online with a Thermo Fisher Scientific Q Exactive HF-X Orbitrap mass spectrometer. Peptide samples were directly injected and separated on an 25 cm-long analytical C18 column (PepSep, 1.9μm3 beads, 75 μm ID) using a 90 minutes long run, starting with a gradient of 7% Buffer B (0.1% Formic acid in 80% Acetonitrile) to 28% Buffer B for 63 min, followed by an increase to 36% in 7 min and a second increase to 95% in 0.5min, and, then kept constant for equilibration at 7% Buffer B for 14.5min. A full MS was acquired in profile mode using a Q Exactive HF-X Hybrid Quadrupole-Orbitrap mass spectrometer, operating in the scan range of 375-1400 m/z using 120K resolving power with an AGC of 3x 106 and max IT of 60ms followed by data independent analysis using 8 Th windows (39 loop counts) with 15K resolving power with an AGC of 3x 105 and max IT of 22ms and a normalised collision energy (NCE) of 26. Each biological sample was analysed in three technical replicates.

LC-MS data analysis

Orbitrap raw data was analysed in DIA-NN 1.8 (Data-Independent Acquisition by Neural Networks)(4) through searching against the reviewed Human Uniprot database containing 27246 proteins (retrieved 4/2021) in the library free mode of the software, allowing up to two tryptic missed cleavages. A spectral library was created from the DIA runs and used to reanalyse them. DIA-NN default settings have been used with oxidation of methionine residues and acetylation of the protein N-termini set as variable modifications and carbamidomethylation of cysteine residues as fixed modification. N-terminal methionine excision was also enabled. The match between runs (MBR) feature was used for all analyses and the output (precursor) was filtered at 0.01 FDR and finally the protein inference was performed on the level of genes using only proteotypic peptides. The generated results were processed statistically and visualised in the Perseus software (1.6.15.0)(5). Values were $\log_2$ transformed, a threshold of 70% of valid values in at least one group was applied and the missing values were replaced from normal distribution. For statistical analysis, Student's t-test was performed, and permutation-based FDR was calculated. Principal Component Analysis (PCA) was performed using Perseus standard settings.

Immunofluorescence and confocal imaging

D30 organoids were fixed for 1hr in 4% (w/v) paraformaldehyde in PBS at room temperature (RT), cryo-protected in 30% (w/v) sucrose in PBS, overnight (O/N), or until they sank, at 4^0C, and embedded in optimal cutting temperature (O.C.T.) compound (Shakura Finetek USA Inc, #4583). O.C.T. embedded organoids were frozen using a dry ice/ethanol bath and stored at -80^0C until further processing. 15μm-thick organoid sections were obtained in a cryostat (Leica cryostat), blocked in 10% (v/v) normal goat-serum (NGS) solution, containing 0.3% TritonX-100/PBS, and incubated O/N in primary antibodies, at 4^0C. All primary antibodies were detected using AlexaFluor secondary antibodies (Invitrogen), incubated for 90min at RT. A detailed list of antibodies can be found in Key Resources Table. 4',6-diamidino-2-phenylindole; DAPI (Abcam, #ab228549) and DRAQ5 (Abcam, ab108410) were used for nuclear staining. Imaging was performed on a LEICA SP5 and a NIKON A1R HD confocal microscope, using a X40 oil-immersion objective, a X20 air objective or a X20 oil-immersion objective (whole slice imaging). For all immunofluorescence experiments a minimum of 3 slices/ organoid were imaged and analysed.

EdU click-it assay

D30 organoids were incubated in 10μM 5-ethynyl- 2'-deoxyuridine (EdU) (Invitrogen, #C-10337, Component A) diluted in cerebral organoid differentiation medium, for 2hrs and subsequently

collected and processed as described previously, for immunofluorescence experiments. EdU click-it assay was performed in organoid cryosections per manufacturer's instructions (EdU kit Invitrogen, #C-10337) followed by immuno-staining for Ki67. The $Edu^{+}/Ki67^{+}$ ratio was measured, and cell cycle length was calculated using the formula $Tc=Ts/(EdU^{+}/Ki67^{+})$ (Tc=cell cycle length, Ts= S phase length). A minimum of 3 slices/ organoid were imaged and analysed.

Immunoblotting

D30 or D60 organoids were transferred from culture plates, briefly washed in ice-cold DPBS (PAN-Biotech, #P04-36500), depleted from Matrigel® using the cell recovery solution and subsequently homogenized in RIPA buffer as in ref.(6), supplemented with protease and phosphatase inhibitors, using a motorised pestle mixer. For each biological replicate, 3-5 organoids were pooled and lysed together. Samples were incubated on ice for 15 min, with occasional vortexing and centrifuged for 20 min at 16 000 g at 4°C. Protein concentration of each sample was determined using the BCA protein assay (Pierce™ BCA Protein Assay, Thermo Fisher Scientific). 40 micrograms of protein per lane were prepared in SDS sample buffer (50 mM Tris pH 6.8, 100 mM DTT, 2% SDS, 10% glycerol, 0.1% bromophenol blue), heated to 95^{0}C for 5min and resolved on polyacrylamide gels. Proteins were transferred to 0.2 μm nitrocellulose membranes (Bio-Rad), blocked for 1 hr at RT in 5% bovine serum albumin (BSA) in TBS-T and incubated with primary antibodies O/N at 4^{0}C. Fluorescent secondary antibodies were used for all immunoblotting experiments. A detailed list of antibodies can be found in Key Resources Table. Blots were imaged using an Azure imaging system (Azure Biosystems) and quantified using the Image Studio Software (Li-COR Biosciences), by measuring the intensity of each protein band. HSC70 or β-Actin was used as loading control. Data are shown as arbitrary units (AU) as a proxy for protein expression, after normalisation to control (for protein phosphorylation: phospho-protein values were divided to normalised total protein values, after subtracting immuno-blot background intensity). For each experiment, values from KO organoids were normalised to the mean of the control group.

RNA sequencing and Bioinformatics Analysis

D30 organoids were transferred from culture plates to 1.5ml tubes and briefly washed in ice-cold DPBS. For each biological replicate, 3-4 organoids were pooled in the same tube. After complete removal of DPBS, organoids were homogenized using QIAshredder homogenizers (Qiagen, #79656) and total RNA was extracted using the RNeasy Micro kit (#74004, Qiagen) per manufacturer's instructions. RNA was suspended in RNase-free water and the concentration and purity of the samples was determined using a Nanodrop instrument (Thermo Fisher Scientific, Nanodrop One C). Library preparation and RNA sequencing were performed as a service by GENEWIZ/AZENTA and sequenced on a Novaseq 6000 instrument (Illumina).

Sequence reads were trimmed to remove possible adapter sequences and nucleotides with poor quality using Trimmomatic v.0.36. The trimmed reads were mapped to the Homo sapiens GRCh38 reference genome available on ENSEMBL using the STAR aligner v.2.5.2b. BAM files were generated in this step. Unique gene hit counts were calculated by using featureCounts from the Subread package v.1.5.2. The hit counts were summarised and reported using the gene_id feature in the annotation file. Only unique reads that fell within exon regions were counted. After extraction of gene hit counts, the gene hit counts table was used for downstream differential expression analysis. Using DESeq2, a comparison of gene expression between the KO-control groups of samples was performed. The Wald test was used to generate p-values and log2 fold changes. Genes with an adjusted p-value<0.05 and absolute log2 fold change>1 were called as DEG for each comparison. A gene ontology (GO) analysis was performed on the statistically significant set of genes by implementing the software GeneSCF v.1.1-p2. The goa_human GO list was used to cluster the set of genes based on their biological processes and determine their statistical significance.

Spatial transcriptomics with GeoMX® and Bioinformatics Analysis

Spatial transcriptomics was performed using the NanoString GeoMx® Digital Spatial Profiler in D30 cerebral organoids. *Tissue preparation*. D30 organoids were transferred from culture plates to pre-chilled DPBS, washed quickly twice and fixed in 10% (v/v) neutral buffered formalin (NBF), at RT for 24 hrs with gentle agitation. On day 2, organoids were washed twice for 30min in DPBS, followed by sequential incubations in sucrose solutions (10%, 20% and 30% w/v), prepared in RNAse free water, on ice for 1-2 hrs or until tissue sank. Organoids were then incubated in 50:50 OCT-30% sucrose solution for 30min on ice, before O.C.T. embedding and freezing in dry ice/ethanol bath. 10μm-thick organoid sections were obtained in a cryostat and stored at -80^{0}C until further processing. *Sections processing*. Fixed frozen slides were equilibrated to RT, washed for 5min in DPBS to remove O.C.T. and baked for 30min at 60^{0}C, in order to prevent tissue detachment from the slides. Slices were then dehydrated in serial incubations in ethanol solutions (50% - 100% v/v), air dried for 15-30min, before rehydration and target retrieval step. Slides were incubated in a preheated Tris-EDTA solution (eBioscience™ IHC Antigen Retrieval Solution - High pH, #00-4956-58) for 15min, at 85^{0}C, followed by proteinase K treatment (at a concentration of 0.1 μg/mL for 15 minutes at 37^{0}C). *In situ hybridization*. Overnight hybridization at 37°C was performed using hybridization probes from the Human Whole Transcriptome Atlas (WTA). The slides then underwent two 5min washes, combining equal parts of 4× SSC buffer and formamide. Post-washing, slides were blocked for half an hour and antibodies were applied to identify morphological markers, assisting in the delineation of specific areas of interest (AOIs). We used PAX6 (Paired box 6) (Cell Signaling Technology, #60433S), NESTIN (Cell Signaling Technology, #33475S) and SYTO83 (GeoMx antibody panel) antibodies as our markers. Following O/N incubation at 4^{0}C, slides were washed 4 times in 2x SSC buffer and incubated with secondary antibodies and nuclei marker Syto83, in Buffer W for 1 hour at room temperature in a humidified chamber. Slides were washed 4 times in 2x SSC buffer and placed in the NanoString GeoMx® DSP instrument.

UV light was targeted onto each AOI to facilitate the release of RNA-ID carrying oligonucleotides from the WTA probes, allowing for their individual collection in distinct wells for every AOI. Subsequently libraries were prepared adding Illumina i5 and i7 dual indexing primers to the oligonucleotide tags during PCR for unique indexing of each AOI. AMPure XP beads (Beckman Coulter) were used for PCR cleanup, and library concentration was determined using a Qubit 3.0 fluorometer (Thermo Fisher Scientific). The quality of libraries was assessed with a Bioanalyzer (Agilent), and sequencing was performed on an Illumina NovaSeq 6000 system.

Gene count data for each AOI was derived from raw .fastq files using the GeoMx® NGS Pipeline. Data analysis was conducted using the GeoMx DSP online analysis platform (Nanostring). Raw reads were trimmed, stitched, aligned, and deduplicated, using standard settings. Differential expression analysis, focusing on the comparisons between PAX6$^-$NESTIN$^+$ and PAX6$^+$NESTIN$^+$ in both control and KO and vice versa, KO versus control in PAX6$^-$NESTIN$^+$ and PAX6$^+$NESTIN$^+$ cells. Permutation q-values (*P*-adjusted) were calculated for robust assessment of statistical significance. DEG were calculated using a cut-off of ±1 $\log_2$ fold-change and log *P* value >1.3. PCA was performed using the Q3 normalised read counts output by the online GeoMx DSP analysis platform. Gene Ontology (GO) analysis was performed with g:Profiler. Top GO categories (Biological Process, Molecular Function and Cellular Compartment) for DEG were ascertained with statistical analysis using g:GOSt (Fisher's one-tailed test).

Scanning Electron Microscopy (SEM)

Scanning electron microscopy (SEM) images were obtained using a JEOL JSM-6510 LV SEM Microscope (JEOL Ltd., Tokyo, Japan) equipped with an X–Act EDS-detector by Oxford Instruments, Abingdon, Oxfordshire, UK (an acceleration voltage of 20 kV was applied). Prior to

SEM analysis, the samples (control and KO organoids) were coated with an Au/Pd thin film (4–8 nm) in a sputtering equipment (SC7620, Quorum Technologies, Lewes, UK).

RT-qPCR

Total RNA was extracted from control and KO iPSCs using TRI reagent (Sigma-Aldrich, #T9424) per manufacturer's instructions. RT-qPCR was performed using the LUNA 1-step RT-qPCR kit (E3005L, NEB) on an AriaMx Real-time PCR system (G8830A, Agilent Tech.). Raw Ct values were normalised to GAPDH using the ΔΔCt method(7). Primer sequences (F: forward, R: reverse) used in this study; *OCT4:* F: 5´-GGAGGAAGCTGACAACAATGAAA-3´, R: 5´-GGCCTGCACGAGGGTTT-3´, *NANOG*: F: 5´-ACAACTGGCCGAAGAATAGCA-3´, R: 5´-GGTTCCCAGTCGGGTTCAC-3´, *CNTNAP2 RT-qPCR:* F: 5´-TGCCTAGAGAGATACCACGGTTACT-3´, R: 5´-TTATATCGTAGCCACATCCCTTCTT-3´, *CNTNAP2 Genotyping 1*: 5´-TCTTCCATTGATTTTGCCATCGACC-3´, R: 5´-CAGGCTCTTAAAAATCAACAGAGGGAAGC-3´, *CNTNAP2 Genotyping 2*: F: 5´-TTTGTAGGACGTGACAGGCTTAGATG-3´, R: 5´-AGGGAAGCCATGGTTACCTTTCC-3´, *GAPDH*: F: 5´-ACCACAGTCCATGCCATCAC-3´, R: 5´-TCCACCACCCTGTTGCTGTA-3´.

Image analysis

Bright field images were acquired on an EVOS™ XL Core microscope (Invitrogen). Quantification of projected surface area of the organoids was performed using Image J software, by outlining the area of the organoid. Fold density measurement in SEM images was performed as previously described (8) using the canny edge detection plug-in Image J. In fluorescent images, cell counting was performed either manually (for EdU assay experiment), using the cell counter tool in Image J or according to ref.(9) instructions for cell counting. The cell fraction for each marker was calculated as a % of the total nuclei measured. For $SOX2^+$ (SRY-box 2) and $PAX6^+$ cell fraction, whole images (20X objective) containing ventricular zone-like structures (VZ - concentric arrangements of columnar epithelial cells around a central cavity, mirroring the structure observed in a neural tube's cross-sectional view) were measured and analysed. For Microtubule-associated protein 2 (MAP2) and Glutamate decarboxylase 1 (GAD1) fluorescent staining measurements, whole slice images were obtained and the MAP2 or GAD1 area fraction of each slice was calculated(9). For the VZ analysis, SOX2 and MAP2 staining was used to manually define the VZ boundaries. Area and perimeter of each VZ was measured using Image J (Analyse/Measure tool). Disorganisation of the VZ was calculated using MAP2 staining as an indicator; a VZ containing $MAP2^+$ cells was scored as disorganised, while absence of $MAP2^+$ cells (clear boundaries) assigned a VZ as organised. For each organoid a minimum of 5 VZ from at least 3 separate slices were measured and analysed.

***Cntnap2* KO mice brain tissue collection**

3–4-month-old *Cntnap2*$^{-/-}$ male mice were obtained from JAX laboratories (Strain #017482). All procedures were in accordance with UK Home Office and University of Edinburgh regulations. Animals were backcrossed for more than 10 generations to C57Bl/6J kground. Food and water were provided ad libitum. Weaning of pups was carried out at postnatal Day 21 (PD21). Mice were then group housed (4-5 littermates per cage) by sex and genotype. Cages were maintained in ventilated racks in temperature (20–21°C) and humidity (~55%) controlled rooms, on a 12-h circadian cycle (7 a.m.–7 p.m. light period). For brain tissue collection, animals were anaesthetized with isoflurane and culled. Brain tissue was rapidly extracted, washed thrice in ice cold PBS, and lysed for immunoblotting in RIPA buffer using a Dounce glass homogeniser.

Statistical Analysis

Experimenters were blinded to genotype during image analysis. All data are presented as mean ± S.E.M. (error bars). No randomisation was performed in this study. Normality was assessed

using the Shapiro-Wilk test (α = 0.05) and statistical significance was set a priori at 0.05 (n.s.: non-significant). N number corresponds to biological replicates (single organoid, or pooled organoids), unless otherwise specified. If multiple observations per research object were collected, we performed nested data statistical analysis (specified in figure legends and Sup. Table 7). Details for statistical tests used are provided within figure legends or the relative methods description and summarized in Sup. Table 7. Sup. Fig. 6 contains raw immunoblot data. Statistical analysis was performed using GraphPad Prism 8.

Study Limitations

We used only one KO iPSC line (generated in XCL1 iPSC; male), which recapitulated most of the forebrain organoid phenotypes, previously shown with patient iPSC lines(10). However, given known issues with human iPSC culture and differentiation variability(11), it is important to study more clones of the same lines or generate additional (e.g. CRISPR/Cas9 mediated) loss-of-function mutants of *CNTNAP2* in e.g. female iPSC and additional lines, other than XCL1, as those in ref.(10). For most experiments we used a minimum of 2 separate batches in the analysis, apart from omics, where only 1 batch was used in all experiments. Bulk RNA-seq was only performed in D30 cerebral organoids. Discrepancies between our model and the patient iPSC-derived organoids(10) regarding organoid size may reflect iPSC differentiation protocol or imaging methodology differences between the two studies.

Supplementary Fig. 1

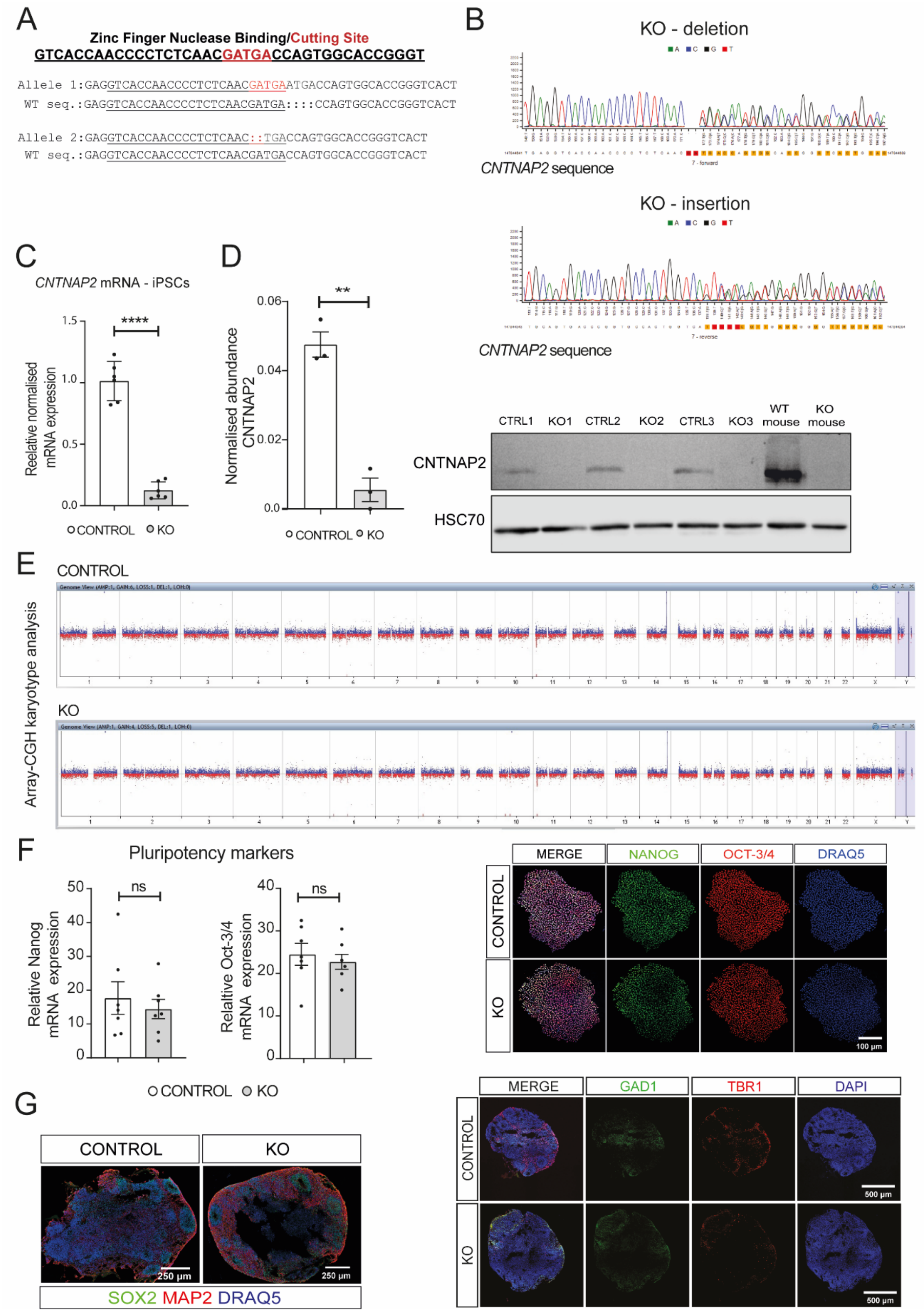

Supplementary Figure 1 IPSC QC and additional data. **A.** Bi-allelic CNTNAP2 KO iPSCs generation with the insertion of 4bp (allele1) and the deletion of 2bp (allele 2), in exon 17, using Zinc Finger Nuclease editing (XCell Science). **B.** Sanger sequencing analysis of the genomic fragment around the two mutation sites, from control and KO iPSCs. **C.** Quantitative RT-qPCR analysis of *CNTNAP2* expression in control and KO iPSCs, showing significantly decreased *CNTNAP2 mRNA* expression (n=2 biological

replicates/genotype and 2-4 technical replicates/genotype. **D.** Western blot analysis that shows CNTNAP2 expression in D30 control organoids, and absence in D30 KO organoids. Each lane corresponds to one biological replicate. **E.** Karyotype analysis of control and KO iPSCs that confirms the normal karyotype of both cell lines. **F.** Quantitative RT-qPCR analysis (left) of the pluripotency markers NANOG and OCT-3/4 expression, and representative fluorescent images (right) from control and KO iPC, confirming the pluripotency of both cell lines. Data points represent different biological replicates collected at different time points. **G.** Representative fluorescent images from D30 control and KO organoids showing the expression pattern of the NPC marker SOX2 and the neuronal marker MAP2 (*left*) and GAD and TBR1 markers (*right*). For C and F, *t*-test analysis, *$P < 0.05$, **$P < 0.01$

See also Table 7

Supplementary Fig. 2

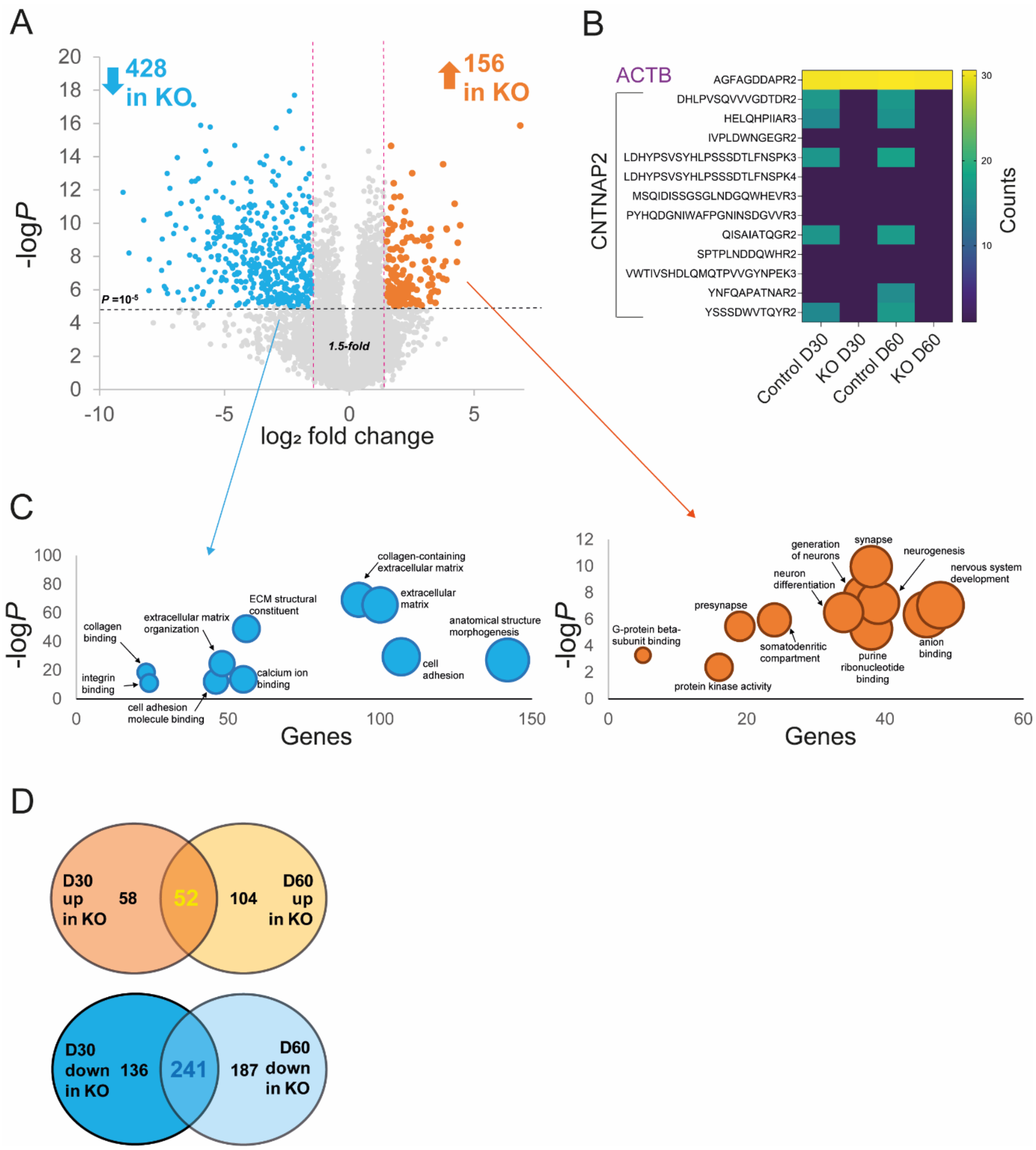

Supplementary Figure 2 Additional Proteomics data A. Volcano plot of D60 organoids proteomics experiment highlighting upregulated and downregulated peptides in KO samples. X-axis demonstrates the log-transformed fold change in abundance (KO/control) and the Y-axis indicates the log-transformed P values associated with individual peptides. A cut-off of ±1.5 fold-change (dashed vertical lines) and P value >10-5 (dashed horizontal line) was applied for differentially expressed peptides. **B.** Heatmap of CNTNAP2 and ACTB peptide counts in D30 and D60 proteomics (KO or control). Numbers in peptide names indicate peptide charge. **C.** GO analysis with g:Profiler for downregulated (left) and upregulated (right) peptides in KO. The Y-axis indicates the log-transformed P values; statistical analysis using g:GOSt (Fisher's one-tailed test).

See also Fig. 2 and Sup. Tables 3, 4, 7

Supplementary Fig. 3

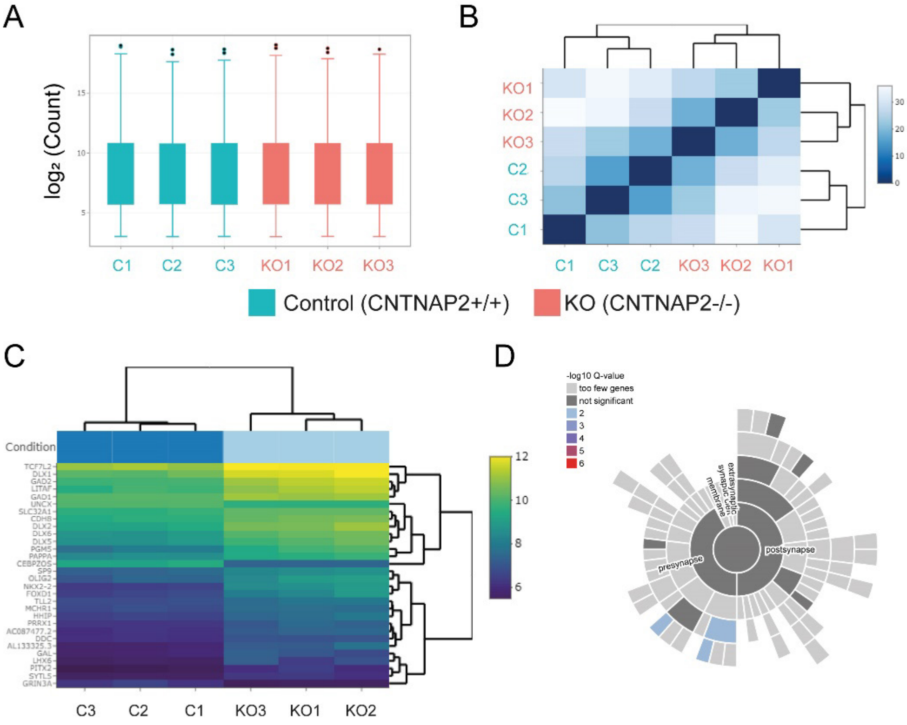

Supplementary Figure 3 Additional RNAseq data A. Normalisation of original RNAseq counts to adjust for various factors such as variations of sequencing yield between samples. These normalised read counts were used to accurately determine differentially expressed genes. **B.** Heatmap of overall similarity among samples assessed by the euclidean distance between samples. This method was used to examine which samples are similar/different to each other. The shorter the distance, the more closely related the samples are. **C.** Bi-clustering heatmap visualising the expression profile of the top 30 DEG sorted by their adjusted *P*-value by plotting their $\log_2$ transformed expression values in samples. This analysis is useful to identify co-regulated genes across the experimental groups. **D.** SYNGO GO analysis of downregulated (left) and upregulated (right) DEG from KO RNAseq analysis of cerebral organoids.

See also Fig. 3 and Sup. Tables 5, 6, 7

Supplementary Fig. 4

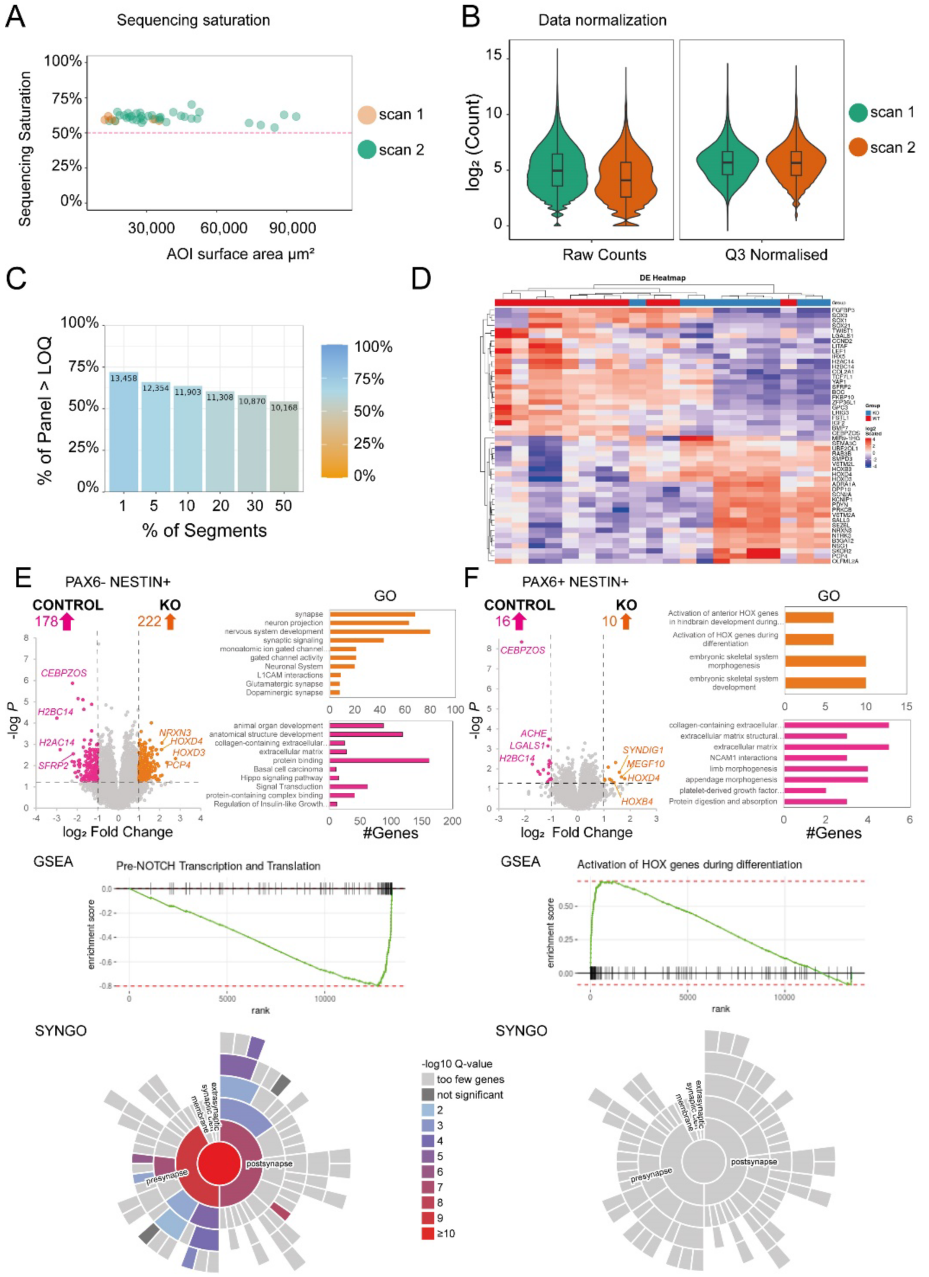

Supplementary Figure 4 Additional spatial transcriptomics data. A. Sequencing quality saturation shown per µm^2, ensuring sensitivity of low expressors. **B.** and **C.** Data was normalised to the third quartile (Q3) to account for differences in cellularity and AOI Size. We analysed 42 AOIs, 0 AOI were below the 50% warning, and we detected 18,676 total targets.13,457 genes normalised by 3rd quartile, expressed above Limit of Quantitation (LOQ) in at least 1% of AOIs. **D.** Heatmap of top 50 DEG sorted by genotype and $\log_2$ fold change. **E.** and **F.** *Left:* Volcano plot of D30 spatial transcriptomics experiment highlighting upregulated and downregulated differentially expressed genes (DEG) in PAX6$^-$/NESTIN$^+$ or PAX6$^+$/NESTIN$^+$ in KO or control samples. X-axis demonstrates the $\log_2$-transformed fold change in abundance in and the Y-axis indicates the negative log-transformed *P*adj (adjusted *P*) values associated with individual mRNAs. A cut-off of ±1 $\log_2$ fold-change (dashed vertical lines) and log *P* value >1.3 (dashed horizontal line) was applied. *Right:* Gene ontology analysis of DEG with g:Profiler. Top categories (Biological Process, Molecular Function and Cellular Compartment) are shown for upregulated and downregulated DEG in the groups depicted. Statistical analysis was carried out using g:GOSt (Fisher's one-tailed test).

See also See also Fig. 4 and Sup. Tables 5, 6, 7

Supplementary Figure 5

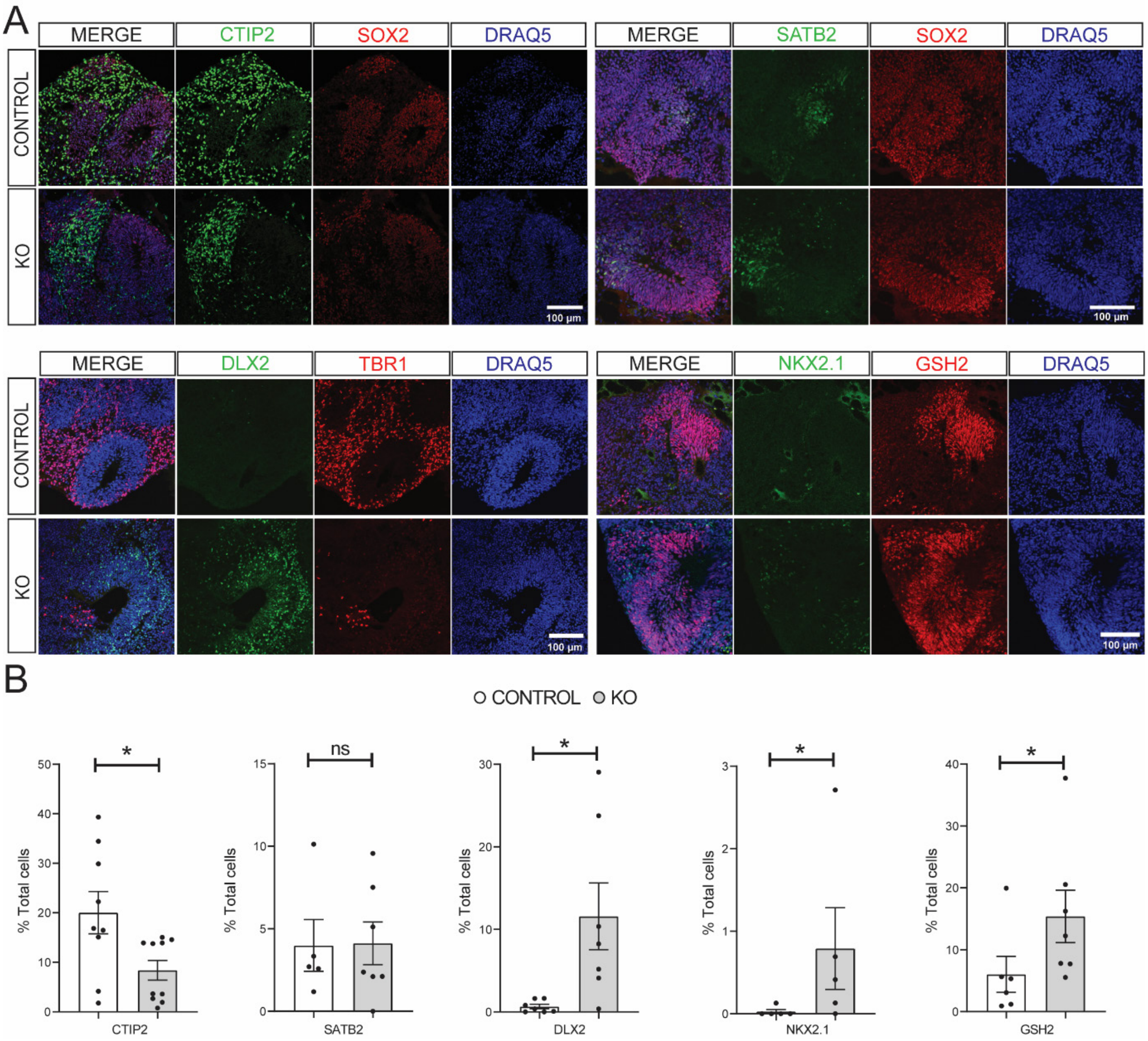

Supplementary Fig. 5 Additional histological data. A. (Top) Representative images from D30 control and KO organoids with cortical layer markers CTIP2 and SATB2 (green) and the neural progenitor marker SOX2 (red) immunostaining. (Bottom) Representative images from D30 control and KO organoids with GABAergic neuron progenitor markers DLX2 (green), NKX2.1 (green) and GSH2 (red) immunostaining.. **B** Quantification of CTIP2$^+$, SATB2$^+$, DLX2$^+$ NKX2.1$^+$ and GSH2$^+$ cell fraction, in D30 cerebral organoids. Total cells were estimated by counting DRAQ5$^+$ nuclei (3-4 separate organoid batches per genotype, n=1- 3 organoids/batch). *Mann Whitney* or Student's *t* test,*P<0.05, CTIP2, COUP-TF-Interacting Protein 2; SATB2, Special AT-Rich Sequence-Binding Protein 2; DLX2, Distal-Less Homeobox 2; NKX2.1, NK2 Homeobox 1; GSH2, Genetic-Screened Homeobox 2

See also Sup. Table 7

Supplementary Table 7 Statistical analysis details

Test	Mean± S.E.M	Standard Deviation (SD)	Significance	Normality- Shapiro Wilk	Parameter	N	Batches /genotype	Descriptive Statistics	Figure
Mann Whitney test	Control: 1±0.037 KO: 0.907±0.029	Control: 0.1652 KO: 0.127	Control vs KO p=0.024	Control; p=0.017 KO; p=0.105	Normalised projected surface area, D30 organoids	Control (19) KO (19)	2	Two-tailed, Mann Whitney U=103.5	**Fig.1D**
Unpaired t test	Control: 1±0.035 KO: 1.031±0.057	Control: 0.152 KO: 0.229	Control vs KO p=0.633	Control; p=0.76 KO; p=0.592	Normalised projected surface area, D60 organoids	Control (19) KO (16)	2	Two-tailed, t=0.4818, df=33	**Fig.1D**
Unpaired t test	Control: 1±0.06 KO: 1.19±0.059	Control: 0.17 KO: 0.156	Control vs KO p=0.043	Control; p=0.76 KO; p=0.043	Normalised EdU/Ki67	Control (8) KO (7)	3	Two-tailed, t=2.238, df=13	**Fig.1F**
2-way ANOVA with Tukey's post-hoc	Control D30: 0.398±0.079 KO D30: 0.732±0.018 Control D60: 0.497±0.051 KO D60: 0.969±0.102	Control D30: 0.211 KO D30: 0.042 Control D60: 0.154 KO D60: 0.288	**Control** D30 vs D60 p=0.78 **KO** D30 vs D60 p=0.206 **D30** Control vs KO p=0.047 **D60**	Control D30 p=0.28 KO D30 p=0.639 Control D60l; p=0.309 KO; p=0.968	Folds density % Area organoids	Control D30 (7) KO D30 (5) Control D60 (9) KO D60 (8)	2	**Organoids age** F(1, 25) = 4.596 p=0.042 **Genotype** F(1, 25) = 26.59 p<0.0001 **Interaction** F(1,25) = 0.791 p=0.382	**Fig. 1H**

			Control vs KO p=0.0004						
Unpaired t test	Control: 20345±1721 KO: 31699±3213	Control: 4866 KO: 9088	Control vs KO p=0.0076	Control; p=0.154 KO; p=0.755	VZ - Area	Control (8) KO (8)	4	Two-tailed, t=3.115, df=14	**Fig. 1J**
Unpaired t test	Control: 548.2±37.53 KO: 663.2±35.02	Control: 112.6 KO: 99.05	Control vs KO p=0.042	Control; p=0.2358 KO; p=0.8079	VZ - Perimeter	Control (9) KO (8)	4	Two-tailed, t=2.223, df=15	**Fig. 1J**
Unpaired t test	Control: 25.09±5.121 KO: 64.83±9.475	Control: 15.36 KO: 26.8	Control vs KO p=0.0017	Control; p=0.934 KO; p=0.67	% Disorganised VZ	Control (9) KO (8)	4	Two-tailed, t=3.809, df=15	**Fig. 1J**
Unpaired t test	Control: 1±0.225 KO: 1.206±0.224	Control: 0.748 KO: 0.809	Control vs KO p=0.527	Control; p=0.058 KO; p=0.082	Normalised Phospho-S6 expression, D30 organoids	Control (11) KO (13)	4	Two-tailed, t=0.642, df=22	**Fig. 5C**
Unpaired t test	Control: 1±0.158 KO: 1.173±0.201	Control: 0.549 KO: 0.699	Control vs KO p=0.506	Control; p=0.074 KO; p=0.256	Normalised Phospho-AKT expression, D30 organoids	Control (12) KO (12)	4	Two-tailed, t=0.676, df=22	**Fig. 5C**
Unpaired t test	Control: 1±0.066 KO: 1.78±0.249	Control: 0.229 KO: 0.864	Control vs KO p=0.01	Control; p=0.192 KO; p=0.193	Normalised Phospho-S6 expression, D60 organoids	Control (12) KO (12)	3	Two-tailed, Welch-corrected, t=3.023, df=12.54	**Fig. 5C**
Mann Whitney test	Control: 1±0.08 KO: 1.297±0.086	Control: 0.267 KO: 0.287	Control vs KO p=0.023	Control; p=0.039 KO; p=0.952	Normalised Phospho-AKT expression, D60 organoids	Control (11) KO (11)	3	Two-tailed, U=26	**Fig. 5C**
Unpaired t test	Control: 42.58±4.229 KO: 41.87±3	Control: 10.36	Control vs KO p=0.898	Control; p=0.974 KO; p=0.939	%PAX6$^+$ cell	Control (6) KO (5)	3	Two-tailed, t=0.131, df=9	**Fig. 5D**

		KO: 6.729			fraction				
Unpaired t test	Control: 46.64±2.4 KO: 47.89±3.7	Control: 7.228 KO: 11.72	Control vs KO p=0.785	Control; p=0.174 KO; p=0.28	%SOX2⁺ cell fraction	Control (9) KO (10)	6	Two-tailed, t=0.276, df=17	**Fig. 5D**
Unpaired t test	Control: 5.926±0.581 KO: 8.977±0.556	Control: 1.745 KO: 1.758	Control vs KO p=0.0015	Control; p=0.304 KO; p=0.793	% MAP2⁺ Area	Control (9) KO (10)	6	Two-tailed, t=3.79, df=17	**Fig. 5D**
Mann Whitney test	Control: 0.699±0.245 KO: 3.373±0.791	Control: 0.734 KO: 2.624	Control vs KO p=0.016	Control; p=0.0026 KO; p=0.306	% GAD1+ Area	Control (9) KO (11)	4	Two-tailed, Mann Whitney U=18	**Fig. 5D**
Mann Whitney test	Control: 18.94±4.468 KO: 8.047±2.623	Control: 13.40 KO: 8.295	Control vs KO p=0.0435	Control; p=0.1987 KO; p=0.02	%TBR1⁺ cell fraction	Control (9) KO (10)	4	Two-tailed, Mann Whitney U=20	**Fig. 5D**
Nested t test	Control: 1.019±0.0713 KO: 0.11±0.034	Control: 0.142 KO: 0.068	Control vs KO p<0.0001	Control; p=0.5 KO; p=0.3	Relative CNTNAP2 mRNA expression	Control (6) KO (6)	3	Two-tailed, t=12.52, df=10, F=156.7, DFn=1, Dfd=10	**Sup Fig.1C**
Unpaired t test	Control: 0.0475±0.036 KO: 0.0055±0.0033	Control: 0.0063 KO: 0.0058	Control vs KO p=0.0011	Control; p=0.105 KO; p=0.828	Normalised CNTNAP2, D30	Control (3) KO (3)	2	Two-tailed, t=8.469, df=4,	**Sup Fig.1C**

One-way ANOVA with Dunnett's post hoc	Control: 17.69±4.8<br>KO: 14.42±2.854<br>Human brain: 1.007±0.004	Control: 12.7<br>KO: 7.55<br>Human brain:0.011	Control vs KO p=0.911<br>Control vs Human brain p=0.035<br>KO vs Human brain p=0.009	Control; p=0.842<br>KO; p=0.957<br>Human brain; p=0.0002	Relative NANOG mRNA expression	Control (7)<br>KO (7)<br>Human brain (7)	3	Brown-Forsythe ANOVA test F*(DFn, DFd)=7.516 (2.000, 9.770), p=0.0106<br><br>Welch's ANOVA test W(DFn, DFd)=15.77(2.000, 8.000) p=0.0017	**Sup Fig.1F**
One-way ANOVA with Dunnett's post hoc	Control: 24.5±2.56<br>KO: 22.74±1.76<br>Human brain: 1.015±0.01	Control: 6.78<br>KO: 4.66<br>Human brain:0.027	Control vs KO p=0.92<br>Control vs Human brain p=0.0003<br>KO vs Human brain p<0.0001	Control; p=0.657<br>KO; p=0.887<br>Human brain; p<0.0001	Relative OCT-3/4 mRNA expression	Control (7)<br>KO (7)<br>Human brain (7)	3	Brown-Forsythe ANOVA test F*(DFn, DFd)=52.99 (2.000, 10.64), p<0.0001<br>Welch's ANOVA test W(DFn, DFd)=108.7 (2.000, 8.000) p<0.0001	**Sup Fig.1F**
Unpaired t test	Control: 0.645±0.277<br>KO: 11.58±4.05	Control: 0.733<br>KO: 10.72	Control vs KO p=0.0355	Control; p=0.056<br>KO; p=0.207	%DLX2$^+$ cell fraction	Control (7)<br>KO (7)	4	Two-tailed, Welch-corrected, t=2.694, df=6.056	**Sup Fig.5**
Mann Whitney test	Control: 0.0257±0.0257<br>KO: 0.789±0.495	Control: 0.057<br>KO: 1.107	Control vs KO p=0.0476	Control; p=0.0001<br>KO; p=0.0412	%NKX2.1$^+$ cell fraction	Control (5)<br>KO (5)	3	Two-tailed, Mann Whitney U=3	**Sup Fig.5**

Mann Whitney test	Control: 6.018±2.901 KO: 15.4±4.225	Control: 7.105 KO: 11.18	Control vs KO p=0.035	Control; p=0.0164 KO; p=0.106	%GSH2$^+$ cell fraction	Control (6) KO (7)	3	Two-tailed, Mann Whitney U=20	**Sup Fig.5**
Mann Whitney test	Control: 20.02±4.273 KO: 8.37±1.974	Control: 12.82 KO: 6.244	Control vs KO p=0.0332	Control; p=0.729 KO; p=0.0063	%CTIP2$^+$ cell fraction	Control (9) KO (10)	4	Two-tailed, Mann Whitney U=19	**Sup Fig.5**
Mann Whitney test	Control: 3.988±1.574 KO: 4.113±1.293	Control: 3.52 KO: 3.421	Control vs KO p=0.755	Control; p=0.034 KO; p=0.442	%SATB2$^+$ cell fraction	Control (5) KO (7)	4	Two-tailed, Mann Whitney U=15	**Sup Fig.5**

References

1. Chan WK, Negro D, Munro VM, Marshall H, Kozić Z, Brown M, et al. (2023): Loss of PAX6 alters the excitatory/inhibitory neuronal ratio in human cerebral organoids. *bioRxiv*.2023.2007.2031.551262.
2. Lancaster MA, Renner M, Martin CA, Wenzel D, Bicknell LS, Hurles ME, et al. (2013): Cerebral organoids model human brain development and microcephaly. *Nature*. 501:373-379.
3. Hughes CS, Moggridge S, Muller T, Sorensen PH, Morin GB, Krijgsveld J (2019): Single-pot, solid-phase-enhanced sample preparation for proteomics experiments. *Nat Protoc*. 14:68-85.
4. Demichev V, Messner CB, Vernardis SI, Lilley KS, Ralser M (2020): DIA-NN: neural networks and interference correction enable deep proteome coverage in high throughput. *Nat Methods*. 17:41-44.
5. Tyanova S, Temu T, Sinitcyn P, Carlson A, Hein MY, Geiger T, et al. (2016): The Perseus computational platform for comprehensive analysis of (prote)omics data. *Nat Methods*. 13:731-740.
6. Chalkiadaki K, Hooshmandi M, Lach G, Statoulla E, Simbriger K, Amorim IS, et al. (2023): Mnk1/2 kinases regulate memory and autism-related behaviours via Syngap1. *Brain*. 146:2175-2190.
7. Livak KJ, Schmittgen TD (2001): Analysis of relative gene expression data using real-time quantitative PCR and the 2(-Delta Delta C(T)) Method. *Methods*. 25:402-408.
8. Li Y, Muffat J, Omer A, Bosch I, Lancaster MA, Sur M, et al. (2017): Induction of Expansion and Folding in Human Cerebral Organoids. *Cell Stem Cell*. 20:385-396 e383.
9. Shihan MH, Novo SG, Le Marchand SJ, Wang Y, Duncan MK (2021): A simple method for quantitating confocal fluorescent images. *Biochem Biophys Rep*. 25:100916.
10. de Jong JO, Llapashtica C, Genestine M, Strauss K, Provenzano F, Sun Y, et al. (2021): Cortical overgrowth in a preclinical forebrain organoid model of CNTNAP2-associated autism spectrum disorder. *Nat Commun*. 12:4087.
11. Volpato V, Webber C (2020): Addressing variability in iPSC-derived models of human disease: guidelines to promote reproducibility. *Dis Model Mech*. 13.
