# Supplementary material for: GABA/Glutamate Neuron Differentiation Imbalance and Increased AKT/mTOR Signaling in CNTNAP2−/− Cerebral Organoids
Source: Biol Psychiatry Glob Open Sci. 2024 Nov 8;5(1):100413. doi: 10.1016/j.bpsgos.2024.100413 (PMC11699409; doi:10.1016/j.bpsgos.2024.100413)

Western Blots

Membranes – Figure 5

D30 cerebral organoids

Phospho - S6 (240/244)

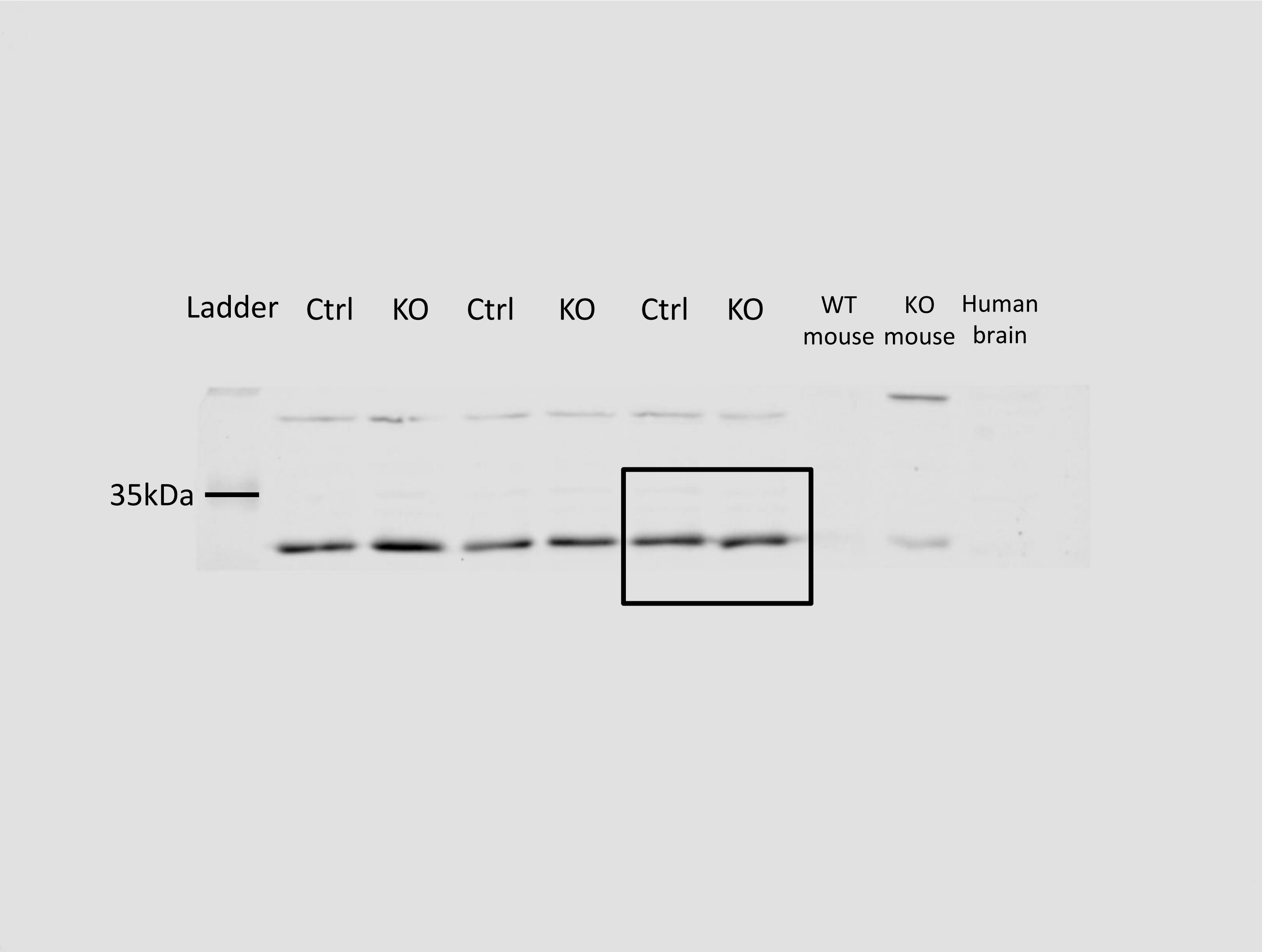

D30 cerebral organoids

Total S6

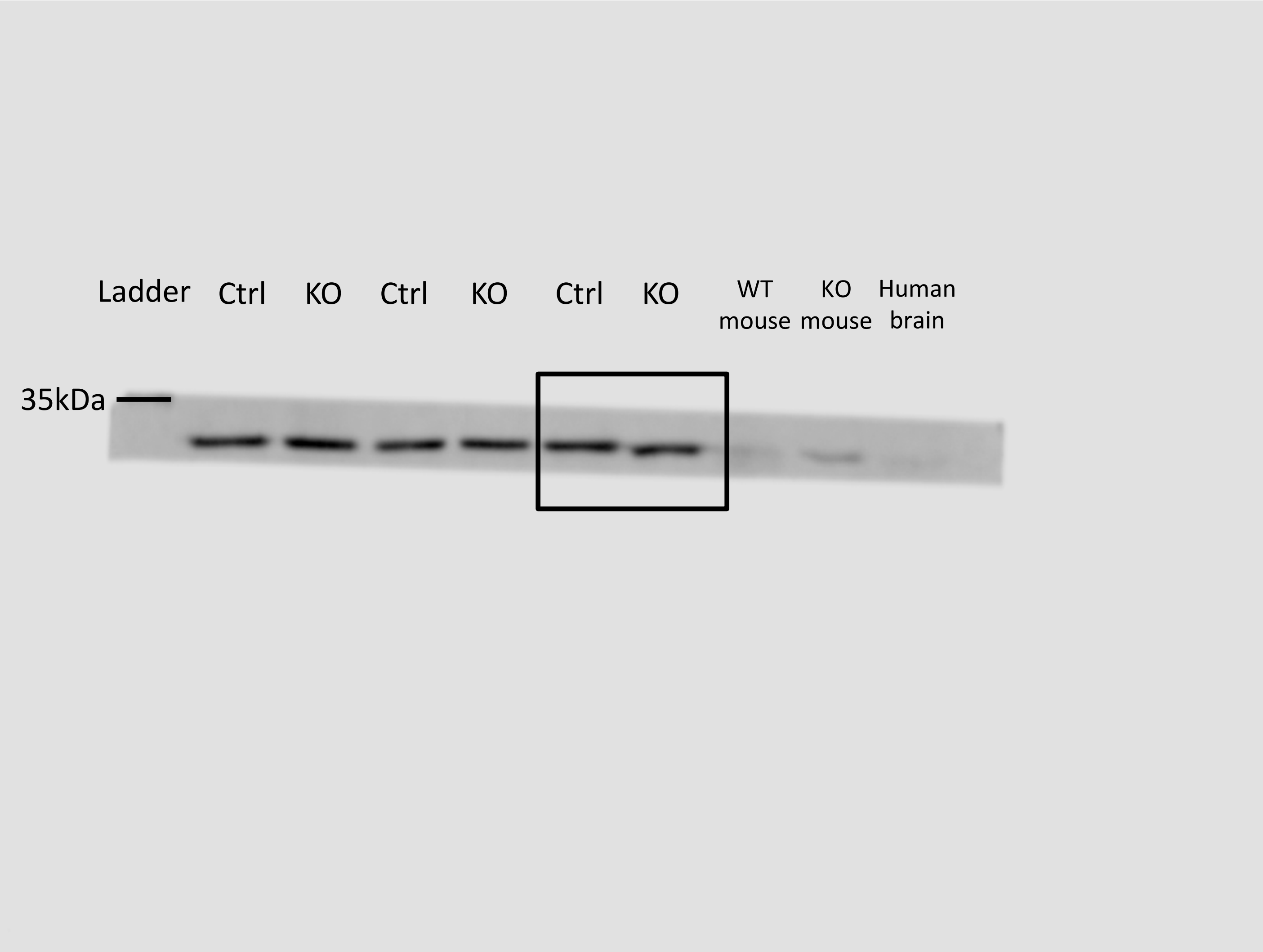

D30 cerebral organoids

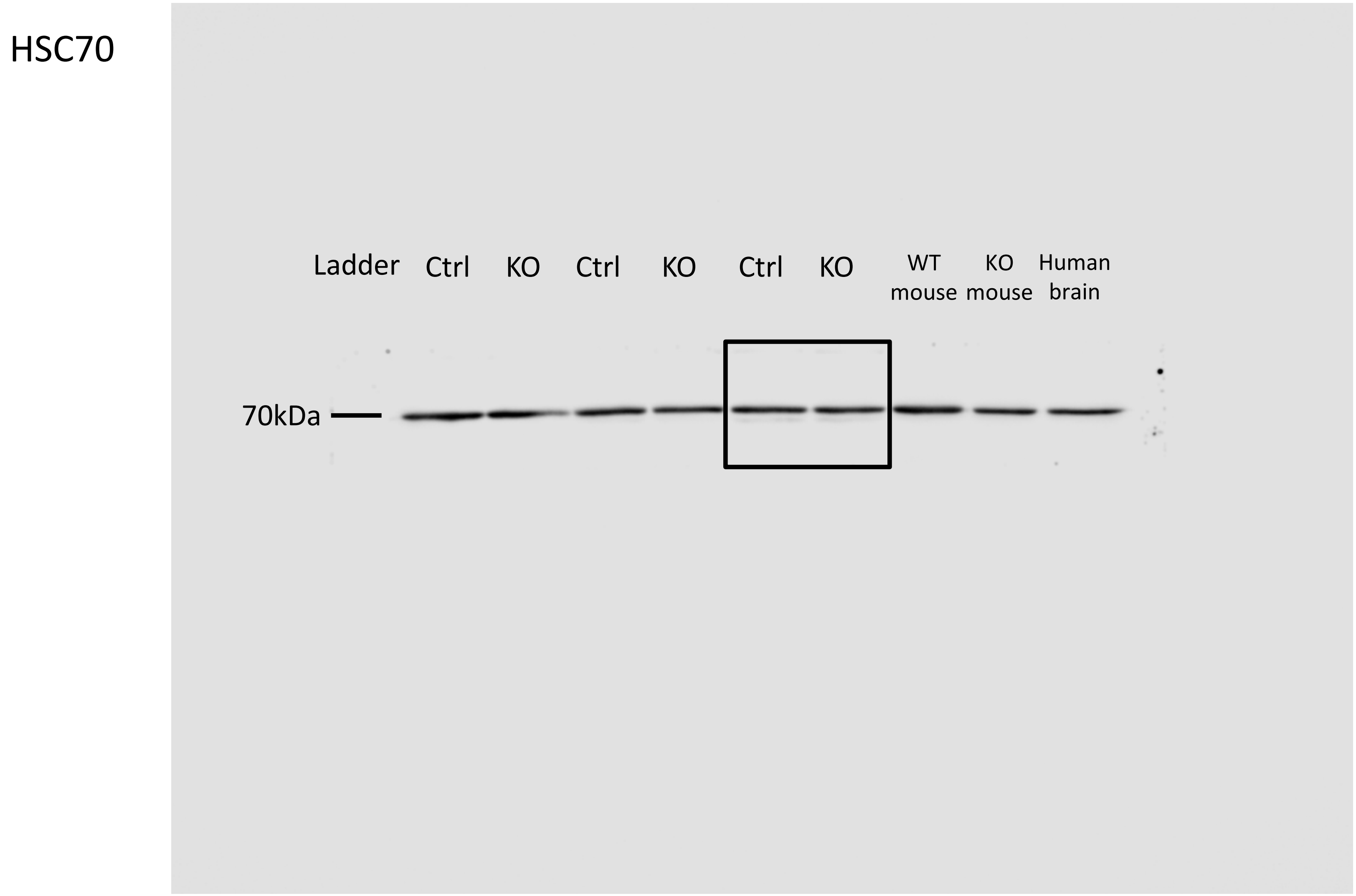

D30 cerebral organoids

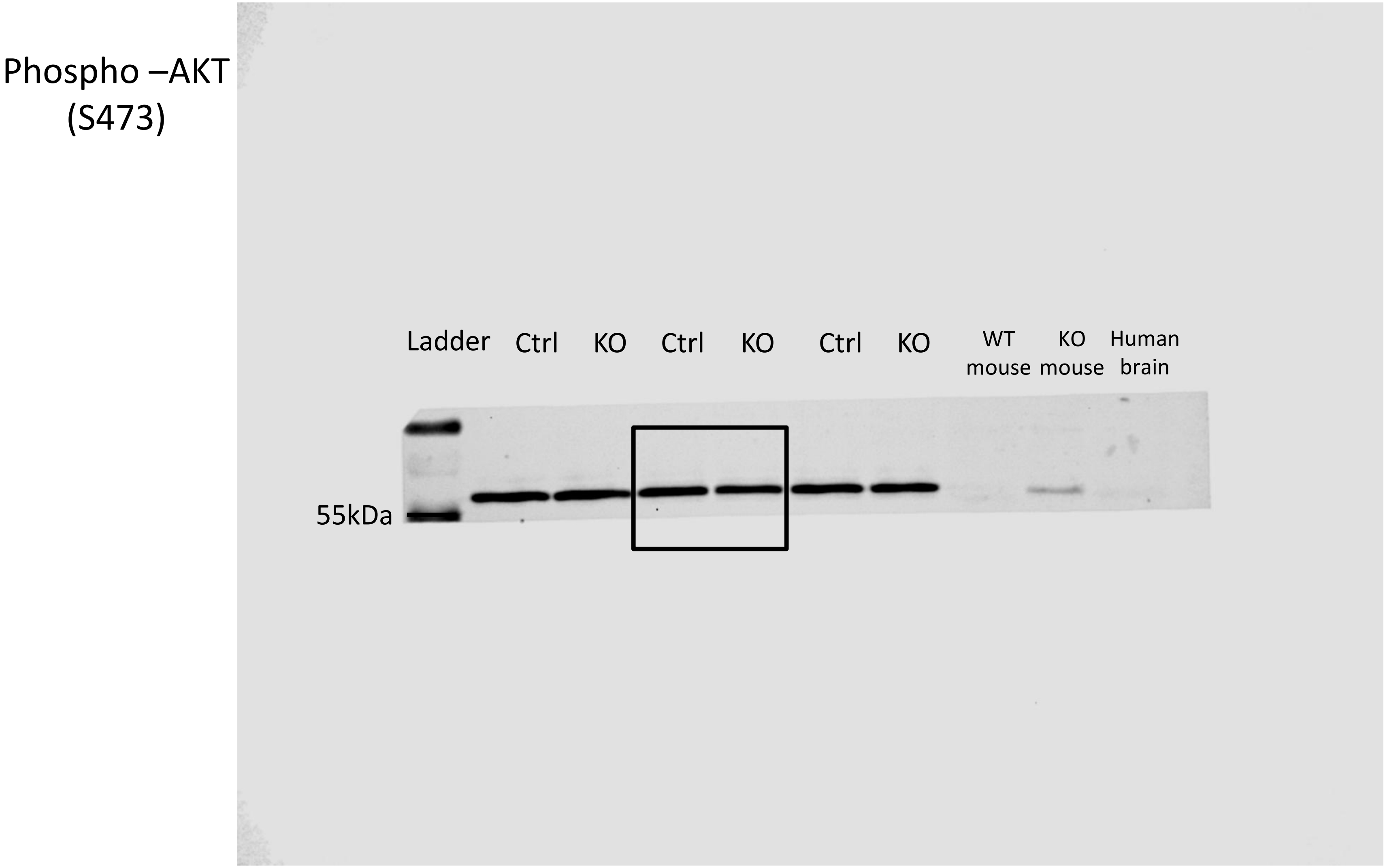

D30 cerebral organoids

Total AKT

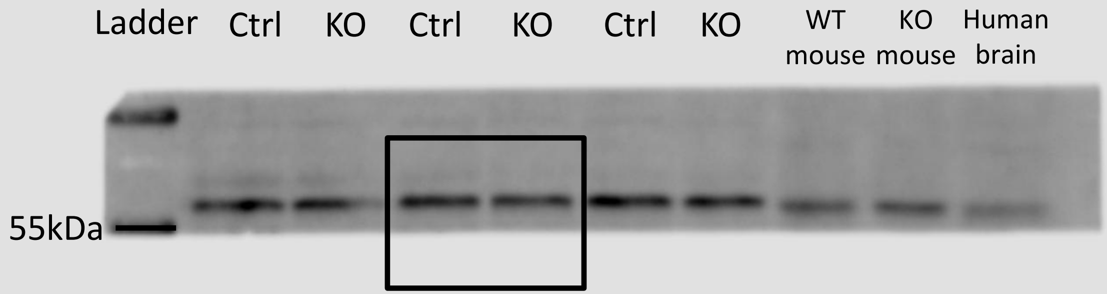

D30 cerebral organoids

HSC70

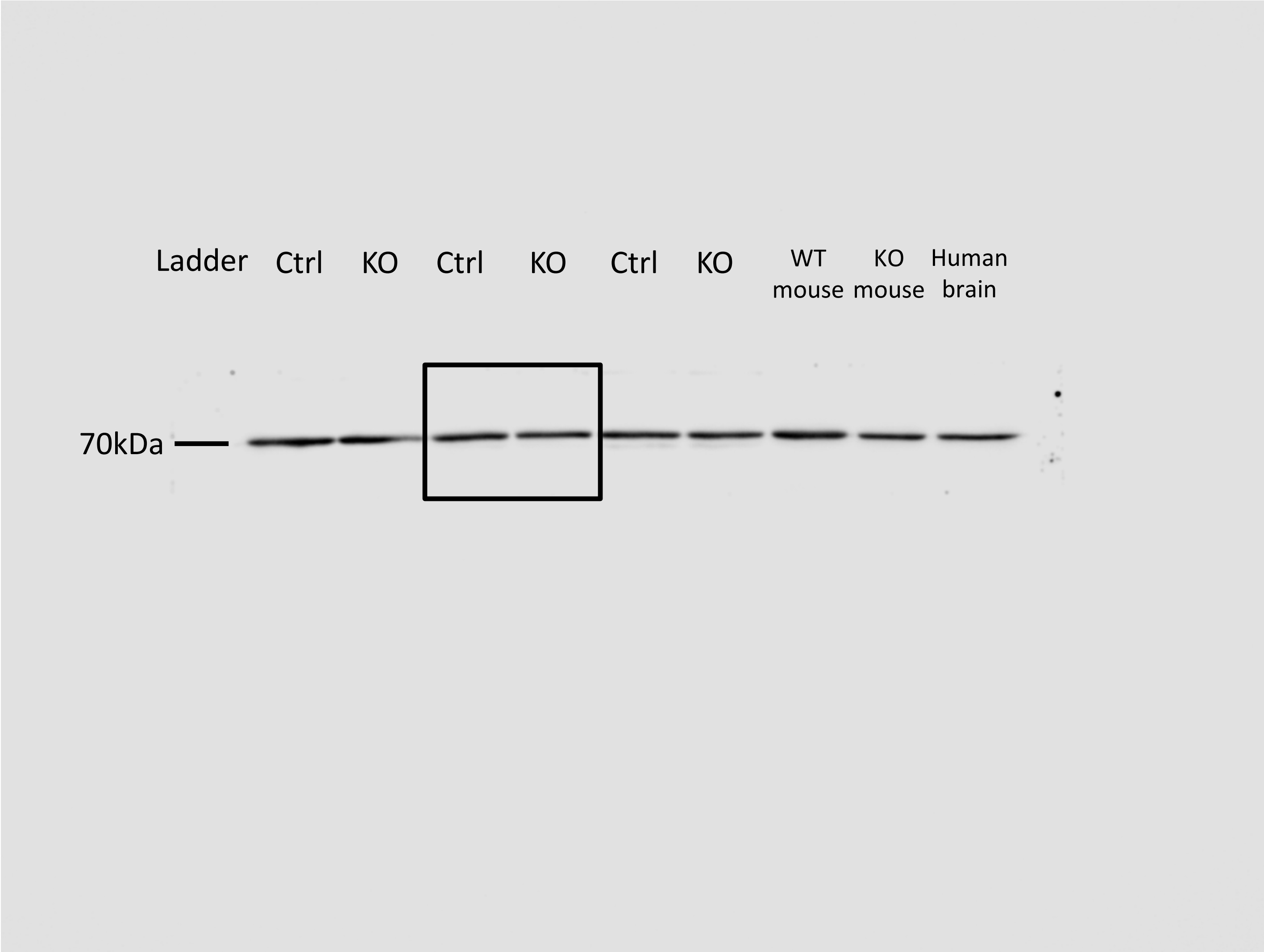

D60 cerebral organoids

Phospho - S6 (240/244)

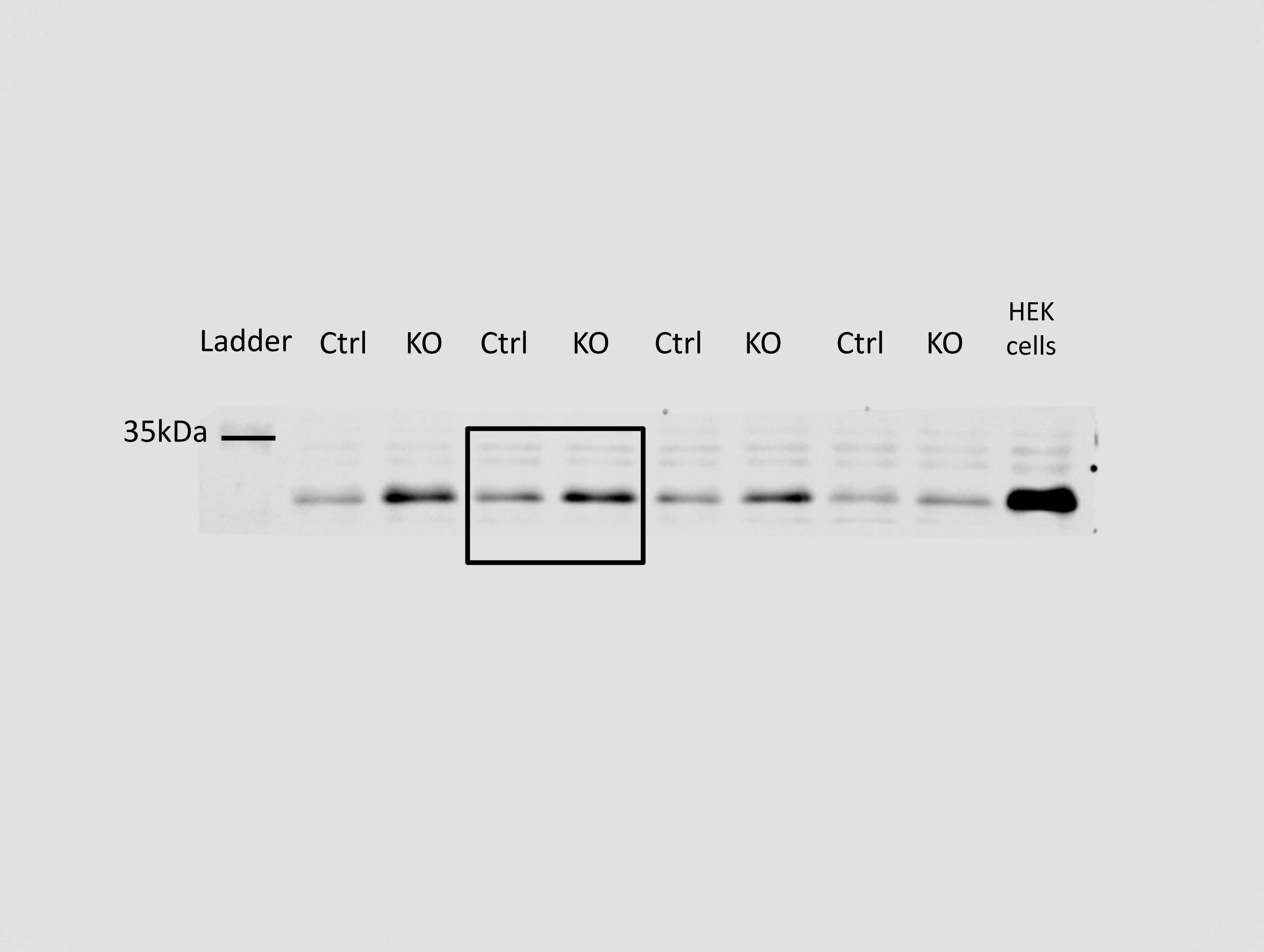

D60 cerebral organoids

Total S6

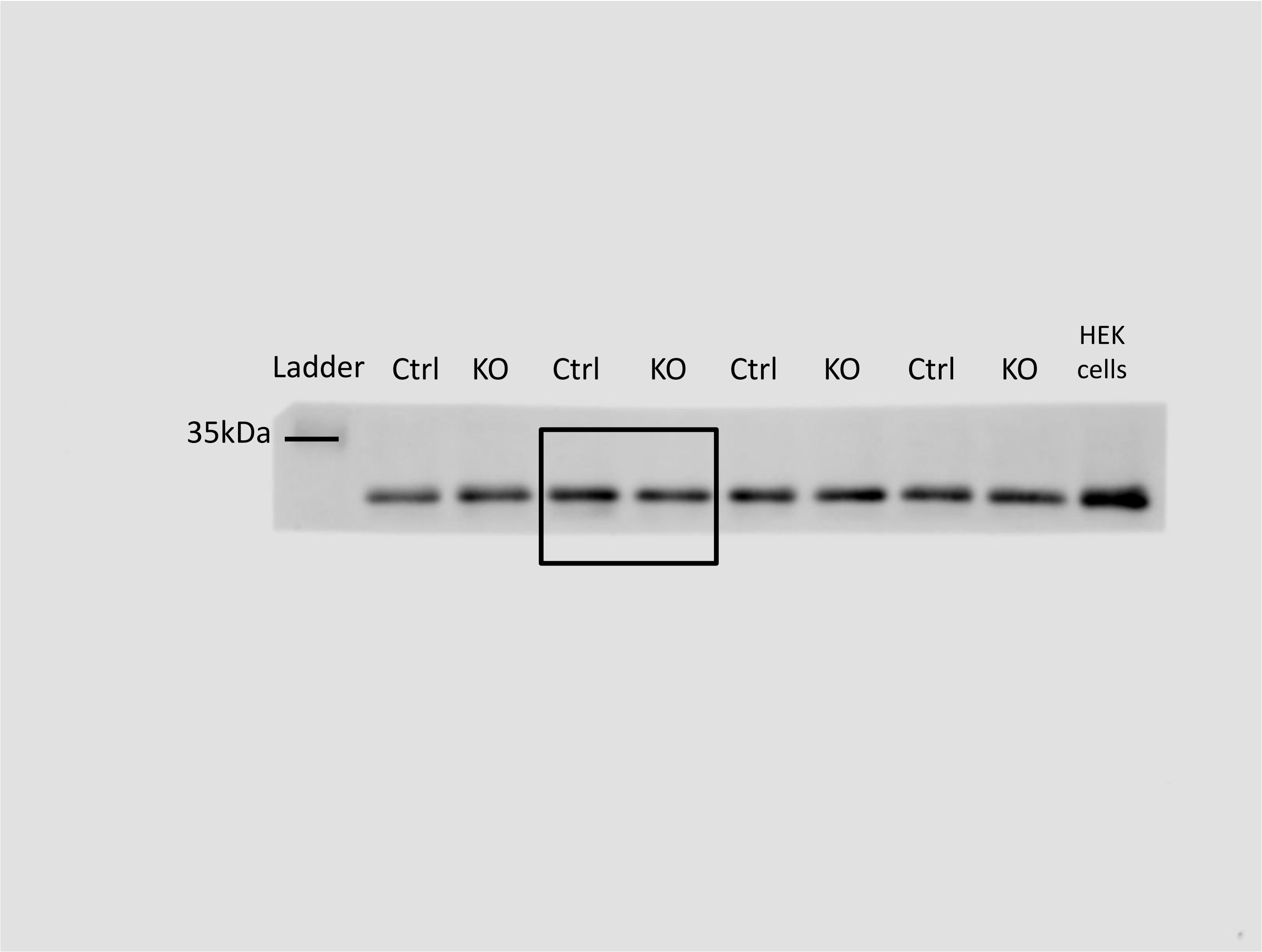

D60 cerebral organoids

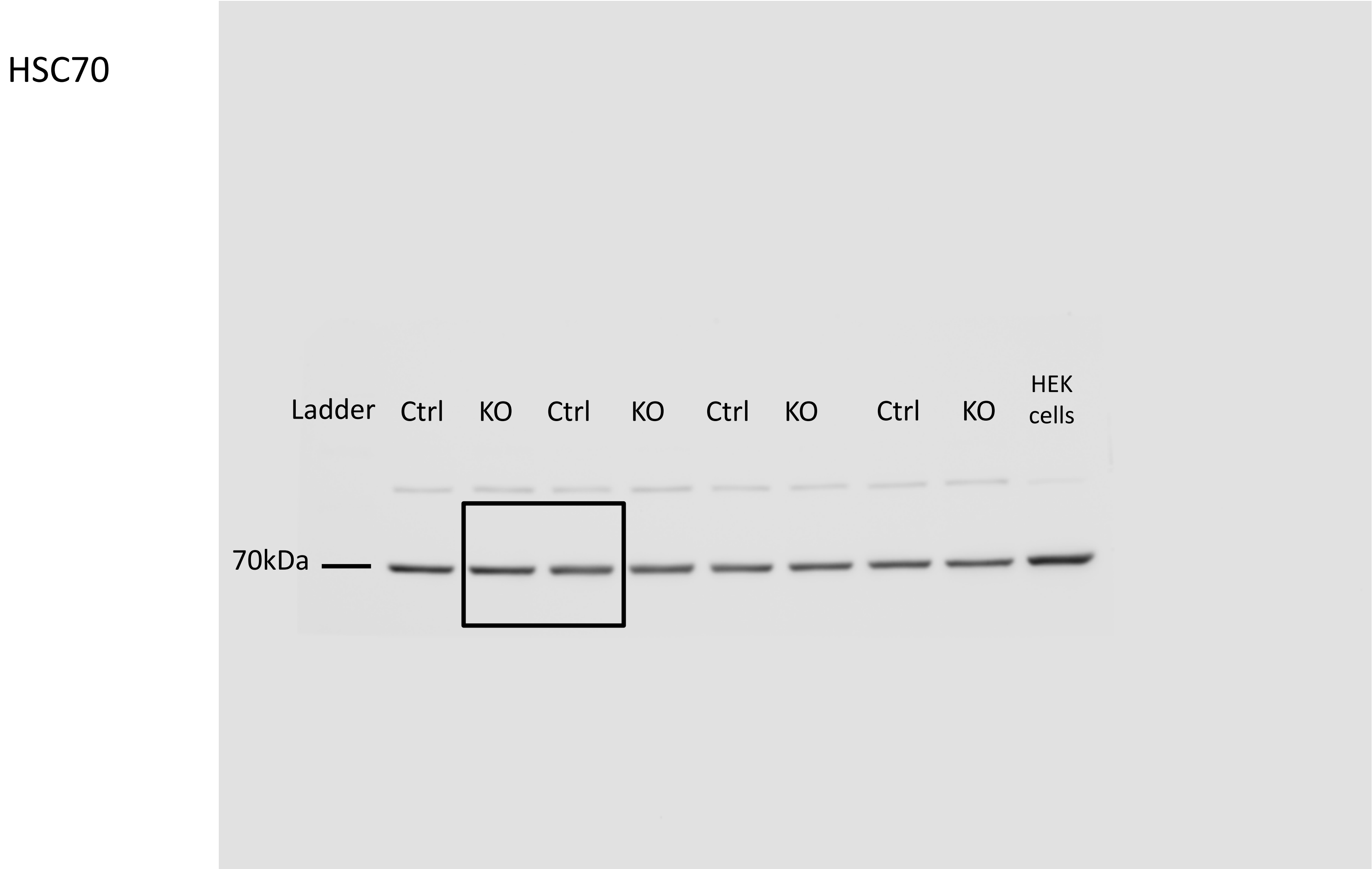

D60 cerebral organoids

Phospho –AKT (S473)

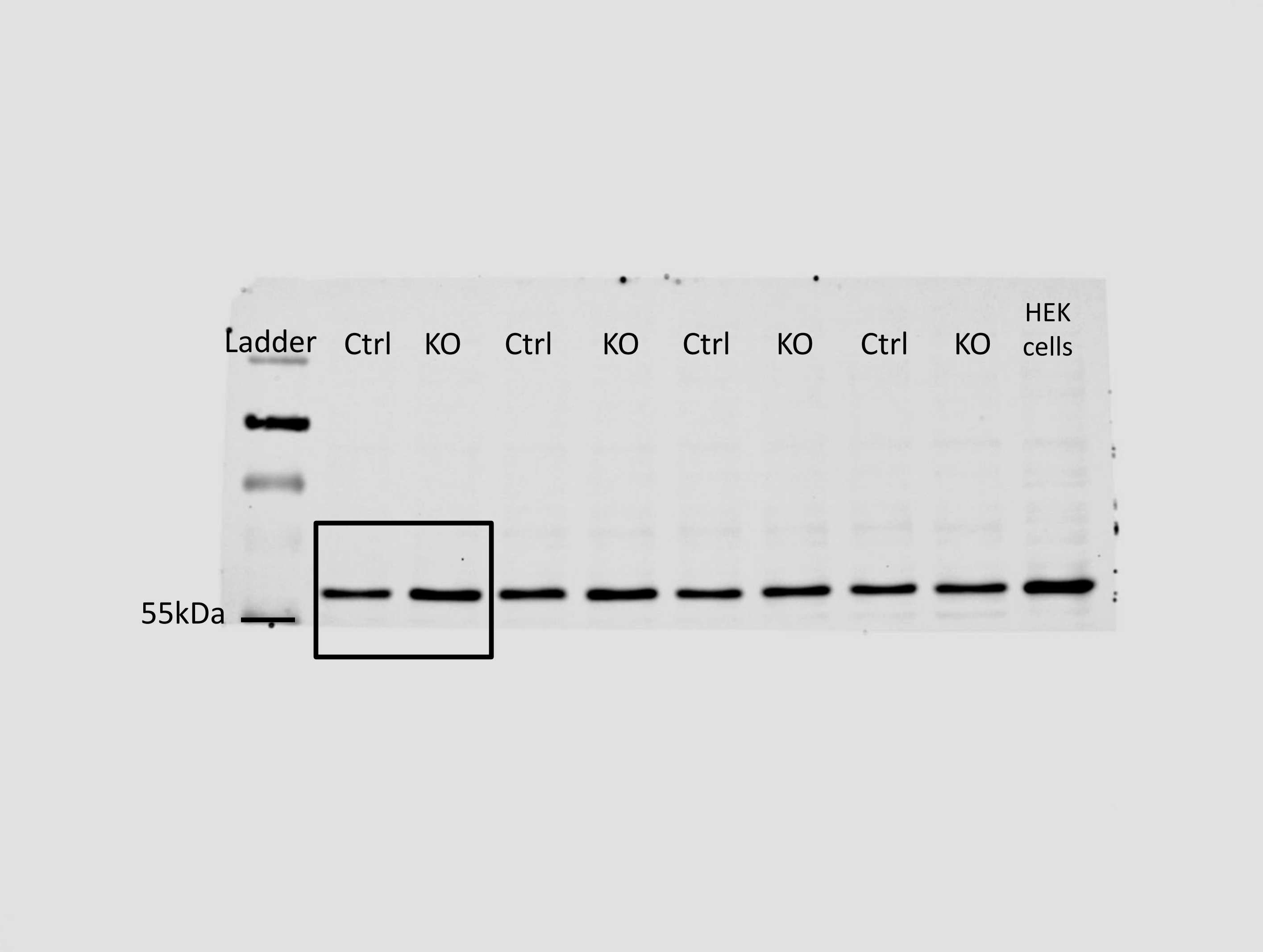

D60 cerebral organoids

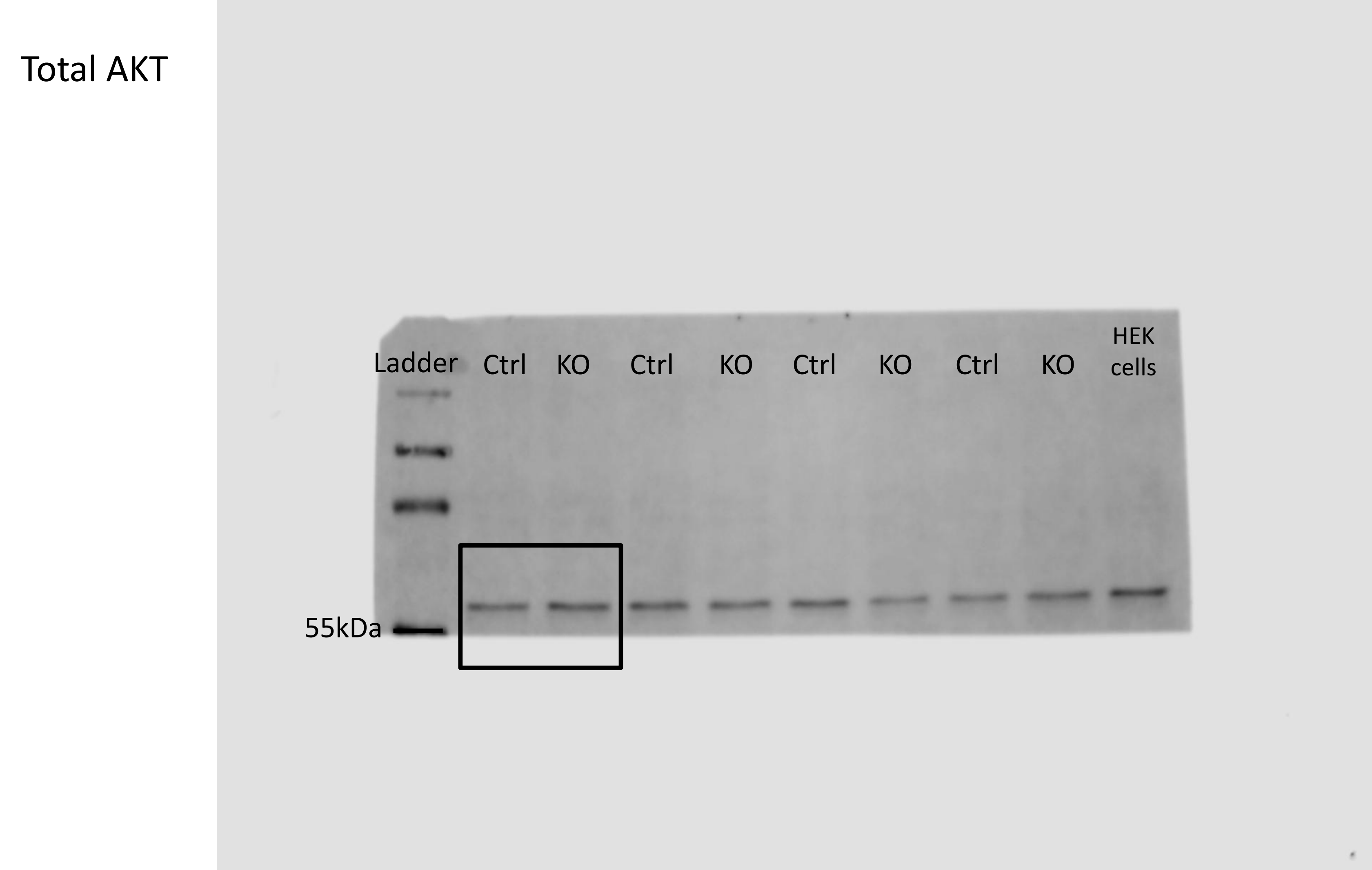

D60 cerebral organoids

HSC70

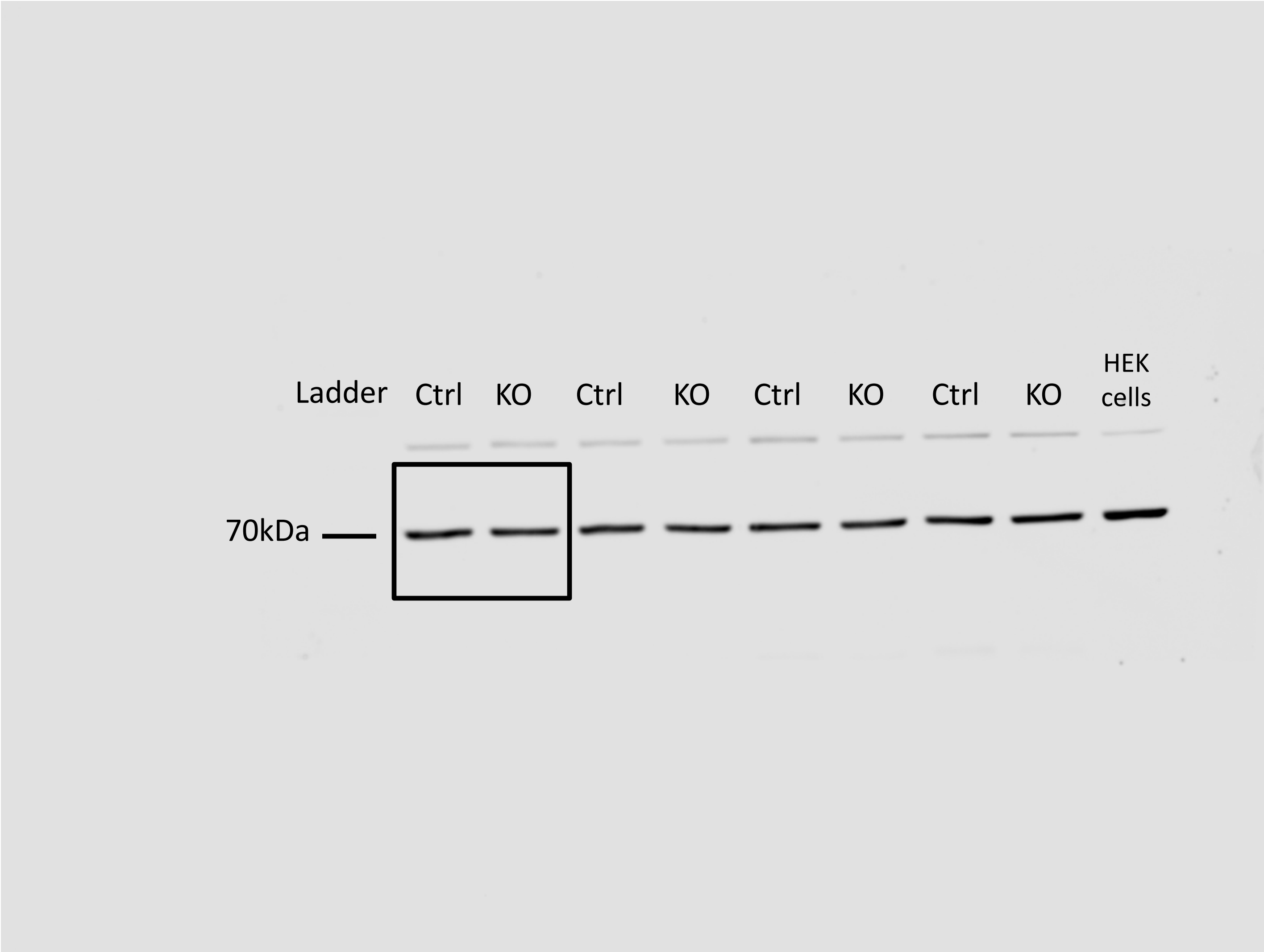

Supplement: Figure S6 [file mmc3.pdf]
